# Biogenic silver nanoparticles synthesized from *Pseudomonas fluorescens*-mediated olive cake waste: antimicrobial, larvicidal activity against *Culex pipiens* and cytotoxicity assessment

**DOI:** 10.1186/s12896-025-01011-2

**Published:** 2025-07-21

**Authors:** Samah H. Abu-Hussien, Muhammad A. Khan, Ammar AL-Farga, Ahmed G. Soliman, Salwa M. El-Sayed, Eslam Adly

**Affiliations:** 1 Department of Agriculture Microbiology, Faculty of Agriculture, Ain Shams University, Cairo, 11241 Egypt; 2 Department of Biological Sciences, Faculty of Sciences, International Islamic University (IIU), Islamabad, Pakistan; 3 Department of Biochemistry, Faculty of Science, University of Jeddah, Jeddah, Saudi Arabia; 4 Department of Agriculture Biochemistry, Faculty of Agriculture, Ain Shams University, Cairo, 11241 Egypt; 5 Department of Entomology, Faculty of Science, Ain Shams University, Cairo, Egypt

**Keywords:** Olive cake hydrolysate, Silver nanoparticles, *Pseudomonas fluorescens*, Larvicidal activity, Antimicrobial nanomaterials, Cytotoxicity, Molecular docking

## Abstract

This study presents an eco-friendly approach for synthesizing silver nanoparticles (AgNPs) using olive cake hydrolysate (OCH), produced through microbial fermentation of olive cake waste by *Pseudomonas fluorescens*. The OCH was analyzed by gas chromatography–mass spectrometry (GC–MS), revealing the biotransformation of olive cake components into bioactive compounds, including 24-norursa-3,12-diene, methyl esters of 9,12-octadecadienoic acid and 9-octadecenoic acid, and α-sitosterol. The biosynthesized olive cake hydrolysate-silver nanoparticles (OCH-AgNPs) were characterized using ultraviolet–visible (UV–Vis) spectroscopy to confirm surface plasmon resonance at 420 nm; Fourier-transform infrared (FTIR) spectroscopy to identify the involvement of hydroxyl and carbonyl functional groups; X-ray diffraction (XRD) analysis to verify the crystalline structure, revealing prominent (111) lattice planes of face-centered cubic (fcc) silver; transmission electron microscopy (TEM) to assess morphology and particle size, showing spherical nanoparticles with an average diameter of 19.6 ± 6.1 nm; dynamic light scattering (DLS) to measure hydrodynamic diameter, yielding a size of 109.8 nm; and zeta potential analysis to determine surface charge, which indicated high colloidal stability with a zeta potential of − 47.0 mV. OCH-AgNPs exhibited superior antimicrobial activity compared to OCH alone, with low MIC values against *P. aeruginosa*, *Candida albicans*, *Aspergillus brasiliensis*, and *Staphylococcus aureus * MRSA. Larvicidal activity, optimized via Box–Behnken design, showed 98.86% mortality of *Culex pipiens* at 1.0 µg/mL (LC₅₀ = 0.40 µg/mL), significantly outperforming OCH (LC₅₀ = 57.22 µg/mL). Histopathological and biochemical analyses of treated larvae revealed structural damage, decreased protein and carbohydrate content, and inhibition of acetylcholinesterase. Cytotoxicity assays on human skin fibroblasts confirmed low toxicity (IC₅₀ >200 µg/mL). Molecular docking identified α-sitosterol as a key bioactive component. These findings underscore the potential of OCH-AgNPs as a sustainable and multifunctional biocontrol agent for microbial and vector management.

**Graphical Abstract:**

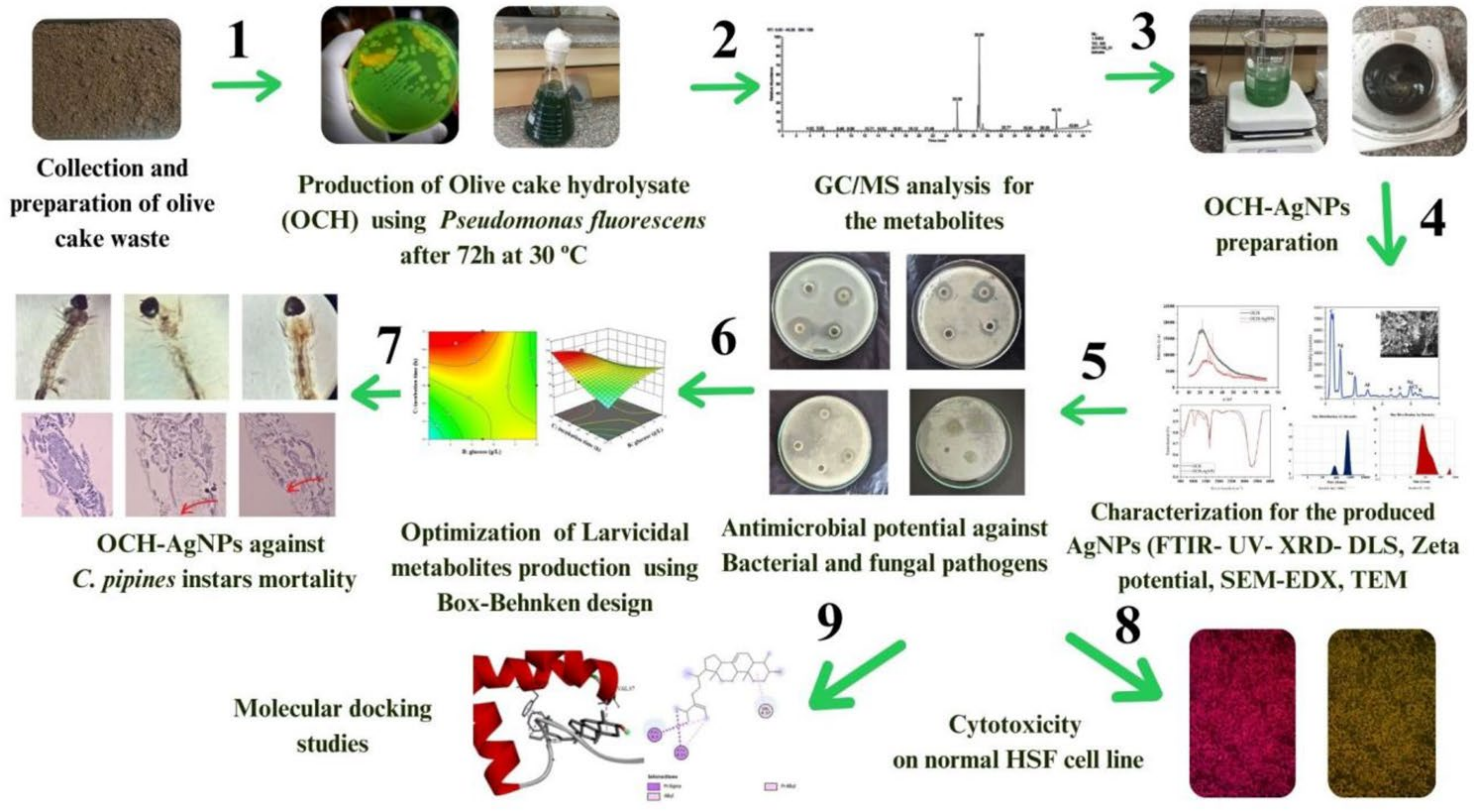

**Supplementary Information:**

The online version contains supplementary material available at 10.1186/s12896-025-01011-2.

## Introduction

Nanoparticles (NPs), due to their nanoscale dimensions (typically 1–100 nm) and high surface-area-to-volume ratio, exhibit physicochemical properties that differ significantly from their bulk counterparts. These include enhanced catalytic activity, improved chemical reactivity, electrical conductivity, and unique biological functions, making them invaluable in a wide range of scientific and industrial applications. Among nanomaterials, metal-based nanoparticles have received substantial attention for their multifunctionality and adaptability [1–3].

Silver nanoparticles (AgNPs) are among the most extensively studied and commercially applied metal nanoparticles, primarily due to their potent antimicrobial [2, 3], antiviral, antioxidant, and insecticidal properties. Their broad-spectrum antimicrobial activity results from multiple mechanisms, including disruption of membrane integrity, reactive oxygen species (ROS) generation, and interference with cellular processes [4]. These properties make AgNPs effective against a wide range of pathogens, including Gram-positive and Gram-negative bacteria and fungi [5]. Consequently, AgNPs are utilized in biomedical applications (e.g., biosensing, drug delivery, wound healing) [6], consumer products (e.g., textiles, food packaging, cosmetics), environmental remediation (e.g., water [7] and air purification) [8], and agriculture (e.g., pesticides, plant protection). The global demand for AgNPs continues to grow, driven by advancements in electronics, catalysis, and biotechnology [9].

Nanoparticle synthesis approaches are broadly categorized into physical, chemical, and biological methods [10]. Physical methods, such as laser ablation and thermal decomposition, offer good control over particle characteristics but require high energy and specialized equipment. Chemical methods, including reduction and sol-gel techniques, are efficient and widely used but often rely on toxic reagents and harsh conditions, raising concerns about environmental and health safety [11].

In contrast, biological or “green” synthesis has emerged as a sustainable alternative, utilizing organisms or their derivatives as natural reducing and stabilizing agents [12]. These methods offer environmental compatibility, biocompatibility, energy efficiency, and cost-effectiveness. Microorganisms, plant extracts, and other biological materials can serve dual roles as reducers and capping agents, leading to nanoparticles with improved stability and biological activity [1].

Recent developments in green nanotechnology have highlighted the potential of microbes in nanoparticle biosynthesis [13]. Bacteria, fungi, actinomycetes, and algae can synthesize metal nanoparticles via extracellular or intracellular pathways, leveraging enzymes, proteins, amino acids, and metabolites to reduce metal ions and functionalize nanoparticle surfaces [14]. Among them, *P. fluorescens* stands out due to its production of bioactive compounds and its plant-protective and antimicrobial properties. Its dual functionality as a biosynthetic and biocontrol agent makes it particularly suitable for synthesizing biologically active nanoparticles [15].

Aligned with green nanotechnology is the increasing focus on sustainable resource utilization and circular bioeconomy principles. Agro-industrial by-products, generated in large quantities worldwide, represent an untapped resource for value-added applications. Olive cake, a lignocellulosic waste from olive oil extraction (∼ 800 kg per ton of olives), is rich in phenolics, sugars, and other bioactive compounds suitable for nanoparticle synthesis. Microbial biotransformation of olive cake can yield hydrolysates that act both as nutrient sources and reducing agents, contributing to waste valorization and nanomaterial production [16, 17].

*C. pipiens*, a primary vector of West Nile virus, lymphatic filariasis, and other arboviruses, poses a significant public health threat worldwide. In recent years, this species has exhibited alarming levels of resistance to conventional insecticides, particularly pyrethroids, carbamates and organophosphates, compromising vector control efforts. According to the latest reports from the World Health Organization (WHO), *C. pipiens* populations across over 60 countries and levels often exceeding 50-fold compared to susceptible strains especially in Africa, Europe, and parts of the Middle East have demonstrated moderate to high resistance levels, with knockdown resistance (kdr) mutations increasingly detected in field isolates. This growing resistance trend underscores the urgent need for novel, eco-friendly alternatives to synthetic insecticides, including nanobiotechnological approaches that offer both targeted efficacy and environmental safety [18–21].

Despite the progress in microbial nanoparticle synthesis, key knowledge gaps remain. These include limited understanding of how biological precursors and synthesis conditions influence nanoparticle characteristics and bioactivity; unclear relationships between microbial metabolites and nanoparticle surface functionalization; lack of safety data on environmental and health impacts; and challenges in scalability and long-term stability under practical conditions.

This study aims to develop an eco-friendly approach for the biosynthesis of silver nanoparticles (OCH-AgNPs) through *P. fluorescens*-mediated biodegradation of olive cake waste. The synthesized nanoparticles are characterized and assessed for their larvicidal activity against *C. pipiens*, antimicrobial activity against selected bacterial and fungal pathogens, and cytotoxicity on normal HSF cells. The synthesis process is optimized using response surface methodology (RSM), and molecular docking is employed to investigate potential mechanisms of antimicrobial and larvicidal action. By integrating microbial biotechnology with agro-industrial waste valorization, the study contributes to sustainable nanoparticle development with potential applications in vector control and antimicrobial formulations.

## Materials and methods

### Collection of olive cake waste

Olive cake waste was obtained from Isis organic company located in Egypt in a previous study [16]. The collected samples were transported to the biology laboratory at the New Programs Administration within Ain Shams University’s Faculty of Agriculture. The samples underwent a drying process at 72 °C for a period of 72 h, after which they were ground into smaller particles. Following preparation, the processed samples were kept at room temperature to be used in subsequent research.

### Microorganisms and media used

*P. fluorescens* used in this study was obtained from a previous investigation and was genetically identified as *P. fluorescens* MICUL B2023. The strain has been deposited in the NCBI GenBank database under accession number OQ729954 (https://www.ncbi.nlm.nih.gov/nuccore/OQ729954) and maintained on King’s B Medium [20]. The antimicrobial properties of the OCH-AgNPs were evaluated against two bacterial strains (*P. aeruginosa* ATCC 27853 and methicillin-resistant *S. aureus*) and two fungal strains (*A. brasiliensis* ATCC 16404 and *C. albicans* ATCC 10231). These test microorganisms were provided by Nawah Scientific in Cairo, Egypt (https://nawah-scientific.com/). The bacterial cultures were grown on tryptone glucose yeast (TGY) medium, while the strains of used fungi were plated on potato dextrose agar (PDA). All cultures were kept at 4 °C until needed for further testing.

### Standard inoculum

A standard inoculum of *P. fluorescens* was prepared by culturing the bacteria in King’s B broth at 30 °C for 24 h with shaking at 120 rpm using rotary shaker (Lab-line Ltd.), yielding a suspension of approximately 7.0 × 10⁵ viable cells/mL for use in experiments [22].

### Gas chromatography-mass spectrometry (GC/MS) analysis

The OCH samples were concentrated through evaporation and then redissolved in methanol before GC-MS analysis. This analysis was performed to identify intermediate metabolites produced during the olive cake biodegradation process by *P. fluorescens*. Chemical profiling was conducted using a Thermo Scientific Trace GC-TSQ mass spectrometer (Austin, TX, USA) with a TG–5MS capillary column (30 m × 0.25 mm, 0.25 μm film thickness). The temperature program started at 50 °C and increased at 5 °C/min to 250 °C (maintained for 2 min), then rapidly rose to 300 °C at 30 °C/min (also held for 2 min). The injector temperature was set at 270 °C, and the MS transfer line was kept at 260 °C. Helium was used as the carrier gas at a steady flow rate of 1 mL/min. After a solvent delay of 4 min, an Autosampler AS1300 automatically injected 1 µL of diluted extract in split mode. Mass spectra were collected using 70 eV ionization energy, scanning across a mass-to-charge range of 50 to 650 in full scan mode, with the ion source maintained at 200 °C. Metabolites were identified by comparing their spectral patterns with reference spectra in the WILEY 09 and NIST 14 mass spectral databases [23].

### Preparation of silver nanoparticles from olive cake waste hydrolysate

A 10 mL aliquot of a 1 mM silver nitrate solution was added dropwise to a glass beaker that contains 20 mL of olive cake hydrolysate that had been previously prepared using *P. fluorescens*. This reaction mixture was then placed in a rotary shaker incubator (Lab-line Ltd.) set at 200 rpm and 30 °C, and left overnight in complete darkness. A control sample consisting of only the OCH without silver nitrate was prepared for comparison. The successful formation of OCH-AgNPs was visually confirmed by a transition in color from pale yellow to light brown [24].

### Characterization analysis of the prepared materials

The biosynthesized OCH-AgNPs were thoroughly characterized using a suite of physicochemical techniques. UV–Visible spectroscopy was conducted using a UV Analyst-CT 8200 spectrophotometer within the 200–800 nm range to detect the surface plasmon resonance (SPR) characteristic of AgNPs. FTIR spectroscopy was performed with a Shimadzu Tracer-100 system in the 1000–4000 cm⁻¹ range (resolution: 4 cm⁻¹) to identify the functional groups involved in nanoparticle reduction and stabilization. Particle size distribution and polydispersity index were determined using DLS with a Zetasizer Nano ZS (Malvern Instruments, UK). Zeta potential measurements were obtained using a NICOMP™ 380 ZLS analyzer (Particle Sizing Systems, USA), which employs phase analysis light scattering, to evaluate colloidal stability at 30 °C. The morphology and surface topography of the nanoparticles were observed using field emission scanning electron microscopy (FE-SEM; JEOL JSM-7800 F, 15 kV). Elemental composition was confirmed by EDX spectroscopy (EDX; VEGA 3-T, 20 kV). Crystallographic structure was analyzed via XRD using a Bruker D2 Phaser (2nd Generation) diffractometer with Cu Kα radiation (λ = 1.5406 Å), and diffraction patterns were collected over a range of Bragg angles. The average crystallite size was estimated using the Debye–Scherrer equation, taking into account the full width at half maximum (FWHM), Bragg angle (θ), and a shape factor (K = 0.9), to assess the nanoscale structural characteristics of the synthesized AgNPs [25, 26]. 

### Antimicrobial potential of OCH-AgNPs

#### Antimicrobial assay using agar well diffusion method

The antimicrobial activity of synthesized OCH-AgNPs was evaluated using the well diffusion assay against *P. aeruginosa* ATCC 27853, methicillin-resistant *S. aureus*, *A. brasiliensis* ATCC 16404, and *C. albicans* ATCC 10231. All microorganisms were cultured on Mueller-Hinton agar (MHA) plates. Each plate was inoculated with 50 µL of microbial suspension (10⁶ CFU/mL). Wells (6 mm diameter) were made using a sterile cork borer, and 0.1 mL of OCH-AgNPs at concentrations ranging from 400 to 25 µg/mL (bacteria) and 1000 to 25 µg/mL (fungi) was added. Gentamycin and distilled water served as positive and negative controls for bacteria, while fluconazole was the positive control for fungi. Plates were incubated at 37 °C for 24 h (bacteria) and at 25 °C for 72 h (fungi). Inhibition zones were measured in centimeters after incubation [23].

### Minimum inhibitory concentrations (MIC), minimum bactericidal concentrations (MBC) and minimum fungicidal concentrations (MFC)

The microdilution method was used to determine the MIC, MBC and MFC of OCH-AgNPs. A stock solution of OCH-AgNPs (400 µg/mL) and control antibiotics (1 mg/mL) **was** prepared and serially two-fold diluted in TGY broth. Standardized microbial suspensions (adjusted to 0.5 McFarland standard) were added to each dilution. After incubation at 37 °C for 24 h, tubes were examined for turbidity to determine MICs. For MBC and MFC, aliquots from clear tubes were sub-cultured onto fresh agar and incubated at 37 °C for bacteria and 25 °C for fungi. The MBC or MFC was recorded as the lowest concentration that killed over 99.9% of the initial inoculum [27].

### Larvicidal potential against *C. pipines* larvae

#### Optimization of larvicidal metabolites of OCH using *P. fluorescens* by box Behnken design

To optimize the production of OCH, The Box-Behnken design [28] employed using three factors, olive cake concentration with low level − 1 (10 g/L), mid-level 0 (15 g/L), high level + 1 (20 g/L), glucose concentration (low 5 g/L, mid 12.5 g/L, high 20 g/L), and incubation time (low 24 h, mid 48 h, high 72 h). To optimize the production of the design matrix (Table 1), 17 predicted trials were experimentally applied to produce 17 OCH supernatant. These supernatants were examined for their larvicidal potential effect on second and third instar larvae of *C. pipiens*. Each treatment was carried out in three replicates. Abbott’s formula was used to calculate the corrected actual mortality percentages as follows: Corrected Mortality (%) = $\:\left\{\frac{\text{T}-\text{C}}{100-\text{C}}\text{x}100\right\},$ T = % mortality in the treatment and C = % mortality in the control.

**Table 1:** Box-Behnken experimental design matrix for optimizing Olive cake waste hydrolysis by *P. fluorescens*

Box-Behnken Design Matrix			
Run	Olive cake (g/L) (A)	Glucose (g/L) (B)	Incubation time(h) (C)
1	+ 1 (20)	+ 1 (20)	0 (48)
2	0 (15)	+ 1 (20)	+ 1 (72)
3	+ 1 (20)	0 (12.5)	+ 1 (72)
4	-1 (10)	0 (12.5)	+ 1 (72)
5	0 (15)	0 (12.5)	0 (48)
6	0 (15)	-1 (5)	-1 (24)
7	-1 (10)	+ 1 (20)	0 (48)
8	-1 (10)	0 (12.5)	-1 (24)
9	0 (15)	+ 1 (20)	-1 (24)
10	0 (15)	0 (12.5)	0 (48)
11	+ 1 (20)	0 (12.5)	-1 (24)
12	+ 1 (20)	-1 (5)	0 (48)
13	0 (15)	-1 (5)	+ 1 (72)
14	0 (15)	0 (12.5)	0 (48)
15	0 (15)	0 (12.5)	0 (48)
16	-1 (10)	-1 (5)	0 (48)
17	0 (15)	0 (12.5)	0 (48)

### Effect of AgNPs on the mortality of *C. pipiens* larvae (second and third instars)

For larval bioassays, twenty-five second and third instar *C. pipiens* larvae were placed in 100 mL beakers containing various concentrations of OCH and OCH-AgNPs. All concentrations were expressed in µg/mL based on the volume of OCH or OCH-AgNP suspension added to the larval medium. Larvae were fed breadcrumbs and maintained at 25–30 °C with a 14:10 h light-dark cycle for 24 h. The time from treatment to larval death was recorded in hours. After each assay, dead larvae were counted, and distilled water served as the negative control. All tests, including controls, were performed in triplicate. Mortality percentages were calculated and adjusted using Abbott’s formula as follows [21, 29]:

$$\begin{aligned}&\:\text{C}\text{o}\text{r}\text{r}\text{e}\text{c}\text{t}\text{e}\text{d}\:\text{m}\text{o}\text{r}\text{t}\text{a}\text{l}\text{i}\text{t}\text{y}\left(\%\right)\cr&=\frac{\%\:\text{t}\text{e}\text{s}\text{t}\:\text{m}\text{o}\text{r}\text{t}\text{a}\text{l}\text{i}\text{t}\text{y}-\%\:\text{c}\text{o}\text{n}\text{t}\text{r}\text{o}\text{l}\:\text{m}\text{o}\text{r}\text{t}\text{a}\text{l}\text{i}\text{t}\text{y}}{100-\%\:\text{c}\text{o}\text{n}\text{t}\text{r}\text{o}\text{l}\:\text{m}\text{o}\text{r}\text{t}\text{a}\text{l}\text{i}\text{t}\text{y}}\times\:100\end{aligned}$$

### Morphological and histopathological analysis of *C. pipiens*

OCH and OCH-AgNPs were prepared as previously described. Samples were placed in 200 mL glass beakers (covered with cotton mesh), each containing 25 mosquito larvae, and maintained at 25 °C for 72 h. All treatments were conducted in triplicate. Morphological changes were examined using a Labomed microscope at 40× and 100× magnifications. Dead larvae were mounted on slides for detailed observation. For histopathological analysis, both control and treated larvae were fixed in 3–5% formalin, dehydrated with ethyl alcohol, cleared with xylene, and embedded in paraplast. Section (7 μm thick) were stained with hematoxylin and eosin, and the mid-gut regions were examined and photographed using a Labomed microscope [21]. 

### Total protein content

Total protein content was quantified using the Pierce™ BCA Protein Assay Kit (Thermo Scientific, Product No. 23225 or 23227). For each assay, 50 µL of protein standard (including blank) or test sample was added to microcentrifuge tubes, followed by 450 µL of distilled water, 100 µL of 0.15% sodium deoxycholate, and 100 µL of 72% trichloroacetic acid. After 10 min of incubation at room temperature, samples were centrifuged at 10,000 rpm for 15 min. The supernatant was discarded, and the pellet was resuspended in 50 µL of 5% SDS (in 0.1 N NaOH). Then, 1 mL of BCA reagent was added, and tubes were incubated at 37 °C for 30 min. Absorbance was measured at 562 nm, and protein content was expressed as the amount per 25 larvae [20].

### Determination of total carbohydrate content

Soluble carbohydrate content was quantified using glucose as the standard. A glucose stock solution (1 mg/mL) was serially diluted (12.5–400 µg/mL) to generate a standard curve. Larvae were homogenized in a sterile mortar and centrifuged at 10,000 rpm for 15 min; the supernatant was collected and diluted 1:1 with distilled water. For analysis, 50 µL of each diluted sample was mixed with 100 µL of 75% sulfuric acid, followed by 200 µL of anthrone reagent (5 mg in 100 µL ethanol + 2.4 mL of 75% sulfuric acid). The mixture was heated at 100 °C for 5 min, then cooled at room temperature for 5 min. Subsequently, 100 µL of each sample was transferred to a 96-well plate, and absorbance was measured at 578 nm using a microplate reader. Measurements were performed in six replicates across three independent experiments, with results expressed as means ± standard deviation [20].

### Acetylcholinesterase (AChE) activity assay

Acetylcholinesterase (AChE) activity was measured using an inhibition assay with donepezil (5 mM) as the positive control. In a 96-well plate, 10 µL of indicator solution (0.4 mM in 100 mM Tris buffer, pH 7.5) was added to each well, followed by 20 µL of AChE enzyme (0.02 U/mL in 50 mM Tris buffer, pH 7.5, containing 0.1% bovine serum albumin). Then, 20 µL of either test sample or standard solution and 140 µL of buffer were added. The plate was incubated at room temperature for 15 min. Afterward, 10 µL of acetylcholine iodide (0.4 mM) was added to each well, and the plate was incubated in the dark at room temperature for 20 min. Absorbance was measured at 412 nm using a microplate reader. Results were expressed as means ± standard deviation [20].

### In vitro cytotoxicity of OCH on human skin fibroblast (HSF)

Human skin fibroblast (HSF) cells were obtained from Nawah Scientific Inc. in Mokatam, Cairo, Egypt. The cells were cultured in Dulbecco’s Modified Eagle Medium (DMEM) supplemented with 10% heat-inactivated fetal bovine serum and antibiotics (100 µg/mL streptomycin and 100 U/mL penicillin) to maintain optimal growth conditions. The cultures were kept in a humidified atmosphere containing 5% CO_2_ at a temperature of 37 °C. To determine cell viability, the MTT assay method was employed [30].

### Molecular docking studies

To investigate the potential interaction of OCH-AgNPs bioactive compounds with key mosquito and microbial target proteins, in silico molecular docking was carried out using a standardized workflow.

### Ligand preparation

Major bioactive compounds identified in the GC-MS analysis of OCH were retrieved from the PubChem database in Structure Data File (SDF) format. Each ligand was subjected to energy minimization using Avogadro version 1.2.0 [31], applying the MMFF94 (Merck Molecular Force Field 94) to achieve stable conformations and optimize geometry. The optimized ligands were then converted to PDBQT format using AutoDockTools (ADT) v1.5.7 for compatibility with AutoDock Vina [32].

### Target protein preparation

Five *Culex pipiens* target proteins, acetylcholinesterase (AChE; UniProt ID: Q86GC8), chitin synthase (ChtSynth; UniProt ID: A0A2R4SD28), ecdysone receptor (EcR), GABA receptor (UniProt ID: A0A1 × 9PRB2), and voltage-gated sodium channel (Na_v_; PDB ID: D2XKJ6), were selected based on their essential roles in insect neural function, molting, and structural integrity. Where crystallographic structures were unavailable, homology models were generated using Swiss-Model or retrieved from AlphaFold Protein Structure Database. All protein structures were prepared using AutoDock Tools by removing water molecules, adding polar hydrogens, assigning Kollman charges, and converting files to PDBQT format, ensuring suitability for molecular docking with AutoDock Vina [33].

### Docking protocol

Molecular docking was conducted using AutoDock Vina v1.1.2. The grid box for each target was centered around the predicted active/binding site, defined either from co-crystallized ligands or conserved catalytic motifs. The grid dimensions were adjusted to enclose the active site fully, typically in the range of 20 × 20 × 20 Å. An exhaustiveness value of 8 was used to ensure reliable sampling of ligand conformations. Each docking run generated nine binding poses, from which the lowest binding energy (ΔG in kcal/mol) was selected for analysis [34].

### Visualization and interaction analysis

Docked protein–ligand complexes were analyzed and visualized using BIOVIA Discovery Studio Visualizer 2020. Key intermolecular interactions including hydrogen bonds, π–π stacking, π–cation interactions, and hydrophobic contacts were identified. The strength and orientation of interactions were considered alongside ΔG values to evaluate binding stability and potential biological activity [20].

### Chemical characterization and reagents 

All characterization analyses were carried out in Nawa scientific labs. (www.nawah-scientific.com*)*, Mokattam branch, Cairo, Egypt. All chemicals and reagents used in the study were of analytical grade and were purchased from Sigma-Aldrich (St. Louis, MO, USA).

### Statistical analysis

All experiments were conducted in triplicate (*n* = 3), and data are presented as mean ± standard deviation (SD). Statistical analysis was performed using one-way ANOVA with a significance threshold of *P* < 0.05, employing OriginPro and Design Expert 12 software. Multiple comparisons were conducted using Tukey’s post hoc test at the same significance level (*P* < 0.05) [35].

## Results

### Gas chromatography–mass spectrometry (GC-MS) analysis

GC-MS analysis of olive cake waste before and after microbial treatment by *P. fluorescens* revealed significant compositional changes, indicating extensive biochemical transformation (Table 2; Figure S1). In the untreated sample, the major constituents were fatty acid methyl esters, predominantly 9-octadecenoic acid methyl ester (oleic acid ME, 55.82%), Palmitic acid methyl ester (Methyl hexadecanoate) (14.02%), and linoleic acid methyl ester (Methyl (Z)-octadec-9-enoate) (12.89%). Other notable compounds included Squalene ((6E,10E,14E,18E)-2,6,10,15,19,23-Hexamethyltetracosa-2,6,10,14,18,22-hexaene) (7.28%) and α-sitosterol (5.31%). Following microbial degradation, the chemical profile shifted substantially. Linolenic acid methyl ester (9,12,15-octadecatrienoic acid ME) emerged as the dominant compound (23.93%), along with high levels of 24-Norurs-3,12-diene (24-Norursa-3,12-diene) (21.96%) and 9,12-Octadecadienoyl chloride ((9Z,12Z)-Octadeca-9,12-dienoyl chloride) (18.83%).

**Table 2:** Comparative GC/MS profile of Olive cake waste before and after microbial treatment with *P. fluorescens*

Retention Time (min)	Compound Name (Untreated)	Area %	Molecular Formula	Retention Time (min)	Compound Name (Treated)	Area %	Molecular Formula
**25.06**	Methyl hexadec-9-enoate (Methyl (Z)-hexadec-9-enoate) (Methyl (Z)-hexadec-9-enoate)	1.61	C_17_H_32_O_2_	**16.70**	6-(3-Isopropenylcycloprop-1-enyl)-6-methylhept-3-en-2-one	0.83	C_14_H_20_O
**25.58**	Palmitic acid methyl ester (Methyl hexadecanoate)	14.02	C_17_H_34_O_2_	**19.41**	τ-Cadinol	2.22	C_15_H_26_O
**28.59**	LinOleic acid methyl ester (Methyl (Z)-octadec-9-enoate)	12.89	C_19_H_34_O_2_	**25.66**	Palmitic acid methyl ester (Methyl hexadecanoate)	7.56	C_17_H_34_O_2_
**28.81**	Oleic acid methyl ester (Methyl (Z)-octadec-9-enoate)	55.82	C_19_H_36_O_2_	**28.70**	Linolenic acid methyl ester	23.93	C_19_H_32_O_2_
**29.33**	Stearic acid methyl ester (Methyl octadecanoate)	3.08	C_19_H_38_O_2_	**28.78**	9,12-Octadecadienoyl chloride ((9Z,12Z)-Octadeca-9,12-dienoyl chloride)	18.83	C_18_H_31_ClO
**44.60**	9,12-Octadecadienoic acid, 2,3-bis(trimethylsilyl)oxypropyl ester (2,3-Bis(trimethylsilyloxy)propyl linoleate)	3.76	C_27_H_54_O_4_Si_2_	**29.35**	Stearic acid methyl ester (Methyl octadecanoate)	5.33	C_19_H_38_O_2_
**40.12**	Squalene ((6E,10E,14E,18E)-2,6,10,15,19,23-Hexamethyltetracosa-2,6,10,14,18,22-hexaene)	7.28	C_30_H_50_	**41.50**	9,12-Octadecadienoic acid, 2,3-bis(trimethylsilyl)oxypropyl ester (2,3-Bis(trimethylsilyloxy)propyl linoleate)	2.07	C_27_H_54_O_4_Si_2_
–	–	–	–	**41.66**	24-Norolean-3,12-diene (24-Norolean-3,12-diene	8.98	C_29_H_46_
–	–	–	–	**42.10**	24-Norurs-3,12-diene (24-Norursa-3,12-diene	21.96	C_29_H_46_
–	–	–	–	**44.73**	α-Sitosterol	5.31	C_29_H_50_O

### Visual and spectral characterization of OCH-AgNPs synthesized via *P. fluorescens*-hydrolyzed olive cake waste

**Fig. 1:**
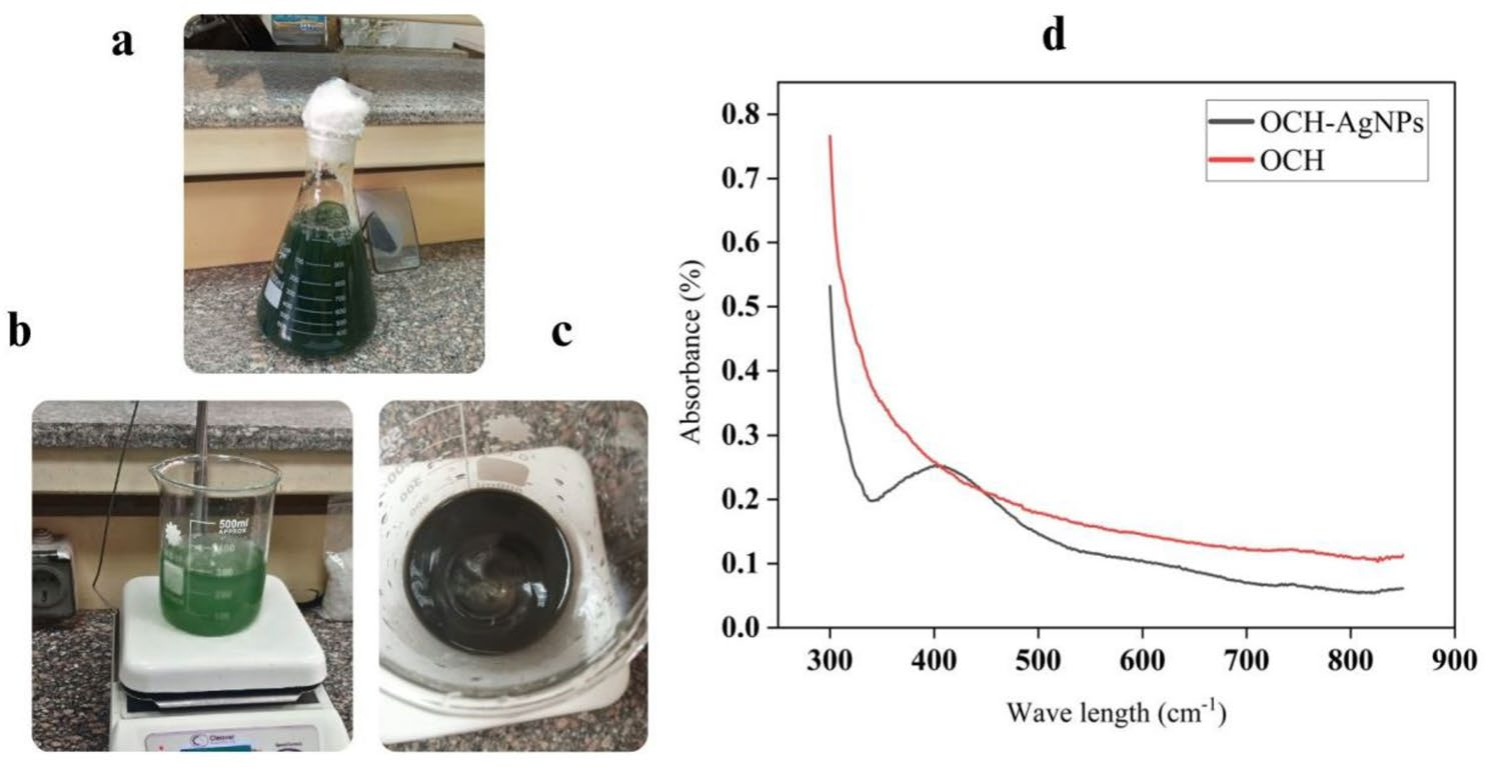
(**a**–**c**) Visual monitoring of AgNP synthesis: (**a**) OCH (olive cake hydrolysate), (**b**) intermediate phase post-AgNO₃ addition, and (**c**) final dark brown OCH-AgNP solution indicating nanoparticle formation. (**d**) UV–Vis spectra of OCH and OCH-AgNPs. The OCH-AgNP spectrum shows a surface plasmon resonance peak at 420 nm, absent in the OCH control

The biosynthesis of silver nanoparticles (AgNPs) using *P. fluorescens*-derived olive cake hydrolysate (OCH) was initially indicated by a distinct color change upon the addition of silver nitrate (AgNO₃). The OCH solution, initially yellowish-green due to microbial metabolites, gradually turned dark brown (Fig. 1). This color change is indicative of the reduction of Ag⁺ ions and is attributed to the excitation of surface plasmon resonance (SPR), a hallmark feature of AgNP formation. Further confirmation was obtained through UV–Visible spectroscopy (Fig. 1d). The OCH-AgNPs exhibited a pronounced absorption peak at  420 nm, consistent with the SPR band typically observed for spherical silver nanoparticles. In contrast, the untreated OCH displayed a broader absorbance centered around 300 nm, likely corresponding to native phenolic or aromatic compounds, but lacked any defined SPR peak.

### Structural and functional group characterization of OCH-AgNPs synthesized via *P. fluorescens*-hydrolyzed olive cake waste

**Fig. 2:**
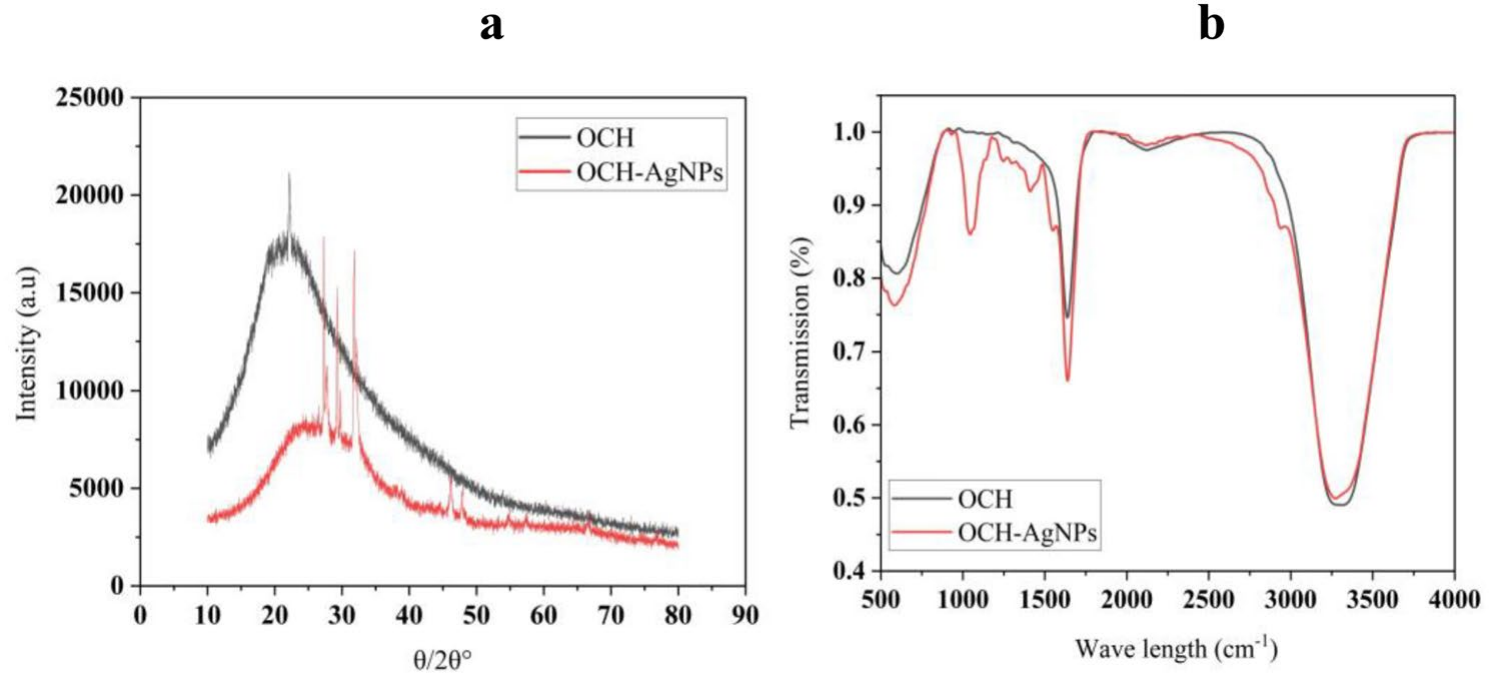
(**a**) XRD pattern of OCH-AgNPs showing characteristic crystalline peaks of silver nanoparticles. (**b**) FTIR spectra of OCH (olive cake hydrolysate) and OCH-AgNPs indicating functional group interactions involved in nanoparticle synthesis

The crystalline nature of the biosynthesized silver nanoparticles (OCH-AgNPs) was confirmed through XRD analysis (Fig. 2). The XRD pattern of the olive cake hydrolysate (OCH) displayed a broad diffraction band centered around 20–30° (2θ), indicative of amorphous organic material. In contrast, the OCH-AgNPs exhibited distinct and sharp diffraction peaks at approximately 2θ = 38°, 44°, 64°, and 77°, which correspond to the (111), (200), (220), and (311) lattice planes of face-centered cubic (fcc) metallic silver, as matched with standard reference data (JCPDS card No. 04-0783; Joint Committee on Powder Diffraction Standards). The presence of these peaks confirms the formation of crystalline silver nanoparticles, with the (111) plane being most intense, consistent with predominant crystal growth along this facet. Complementary characterization using FTIR spectroscopy (Fig. 2) provided insight into the functional groups involved in nanoparticle formation and stabilization. In the OCH spectrum, a broad band between 3300 and 3500 cm⁻¹, corresponding to O–H stretching vibrations, was significantly diminished in OCH-AgNPs, indicating the involvement of hydroxyl groups in silver ion reduction. The aliphatic C–H stretching vibrations near 2900 cm⁻¹ remained largely unchanged, suggesting minimal involvement of these groups. Notably, a shift in the C = O stretching band from approximately 1650 cm⁻¹ in OCH to a lower wavenumber in the OCH-AgNP spectrum suggests interaction or coordination between silver ions and carbonyl-containing moieties. Additional spectral changes in the 1000–1500 cm⁻¹ region, potentially associated with C–O or C–N stretching vibrations, further indicate molecular rearrangements linked to nanoparticle formation.

### Morphological and elemental characterization of OCH-AgNPs synthesized via *P. fluorescens*-hydrolyzed olive cake waste

**Fig. 3:**
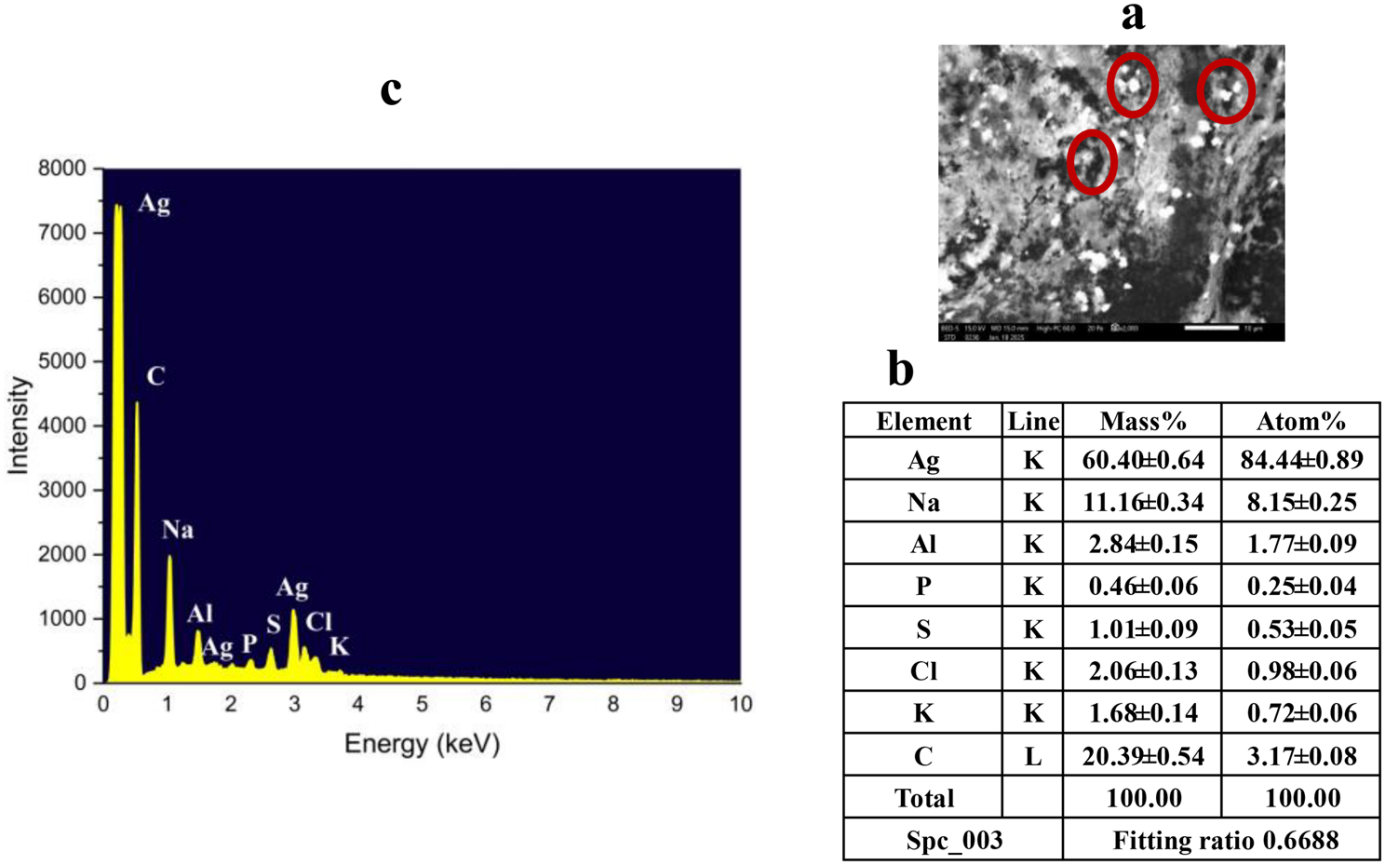
(**a**) SEM image of OCH-AgNPs at 2000× magnification. The bright clusters indicate silver deposition. (**b**-**c**) EDX spectra confirming elemental composition of OCH-AgNPs, including silver and bio-organic elements from OCH (olive cake hydrolysate)

Elemental and morphological analyses were performed to verify the successful formation and surface distribution of silver nanoparticles (AgNPs) on the olive cake hydrolysate (OCH) matrix. EDX analysis (Fig. 3b–c) confirmed the presence of elemental silver, evidenced by a prominent signal at approximately 3 keV, characteristic of metallic Ag. Silver accounted for 20.39 weight% (wt%), validating its substantial incorporation into the nanoparticulate system. Additional peaks corresponding to carbon (C), sodium (Na), aluminum (Al), phosphorus (P), sulfur (S), chlorine (Cl), and potassium (K) were observed. These elements are attributed to residual organic and inorganic constituents from the OCH matrix, which likely contributed to the reduction and stabilization of silver ions during biosynthesis. SEM provided visual evidence of morphological transformation following nanoparticle formation (Fig. 3a). The SEM micrograph of OCH at 2000X magnification (scale bar: 100 nm) showed a more heterogeneous and textured morphology, with noticeable bright clusters distributed across the surface at the same magnification but observed at a smaller scale bar (10 nm). These clusters are consistent with silver nanoparticle agglomerates, suggesting successful deposition and possible surface anchoring onto the hydrolysate matrix.

### Size and surface charge characterization of OCH-AgNPs synthesized via *P. fluorescens*-hydrolyzed olive cake waste

**Fig. 4:**
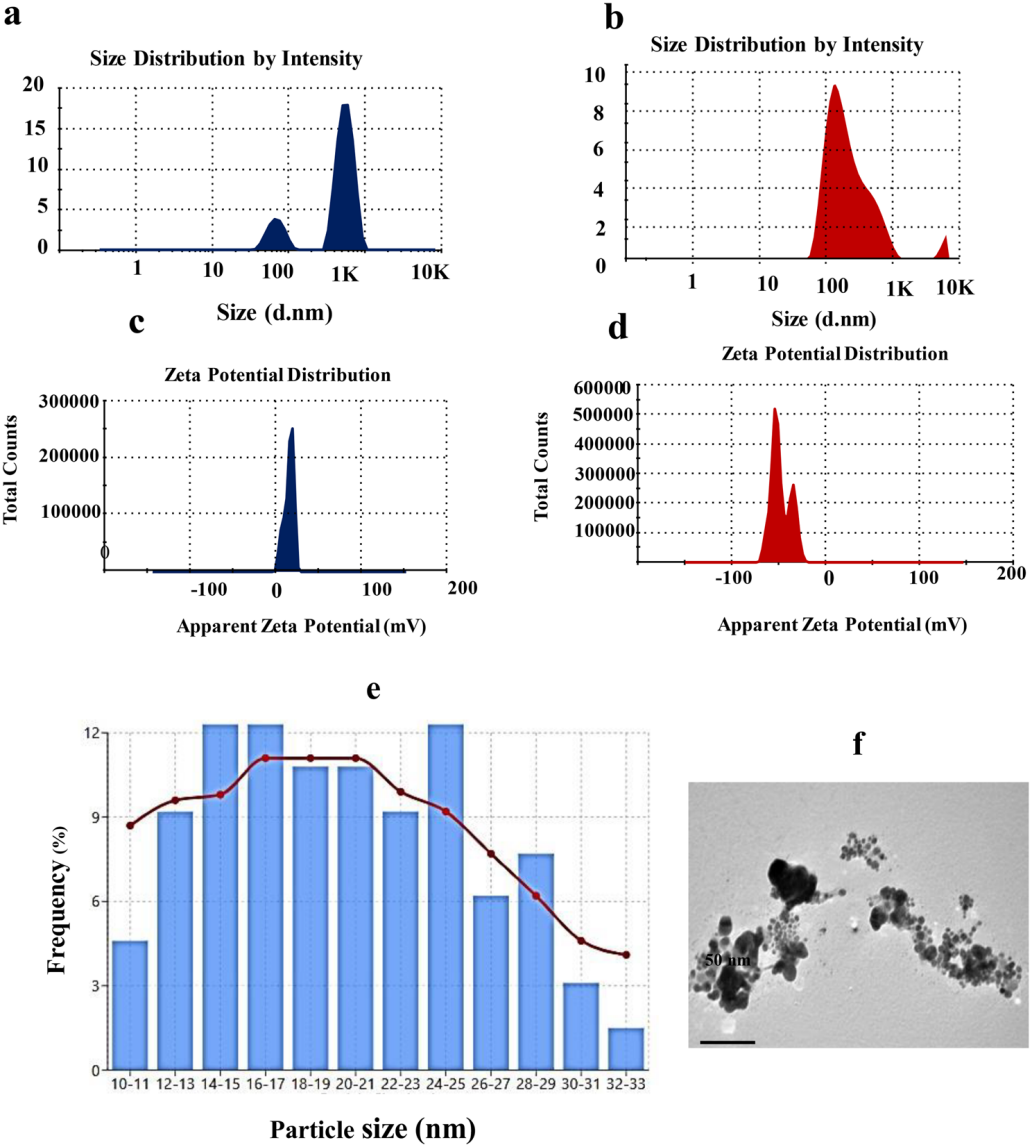
(**a**) DLS size distribution of OCH (olive cake hydrolysate) showing large biomolecular aggregates. (**b**) DLS size distribution of OCH-AgNPs showing a sharp nanoparticle peak at 109.8 nm. (**c**) Zeta potential analysis comparing OCH (+ 11.4 mV) and OCH-AgNPs (− 47.0 mV), indicating improved colloidal stability. (**d**) TEM micrograph showing spherical morphology of OCH-AgNPs. (**e**) Histogram of particle size distribution measured by TEM, showing a modal diameter of 14–15 nm

The particle size distribution and surface charge characteristics of the synthesized silver nanoparticles (OCH-AgNPs) were assessed using DLS, zeta potential analysis, and TEM (Fig. 4). DLS analysis revealed a significant size reduction following nanoparticle formation. The olive cake hydrolysate (OCH) sample exhibited a broad size distribution with a dominant peak at 894.8 nm, consistent with large biomolecular aggregates. In contrast, the OCH-AgNPs sample showed a sharp, unimodal peak centered at 109.8 nm, indicating the formation of smaller, more uniform nanoparticles with lower polydispersity. Zeta potential measurements demonstrated a substantial shift in surface charge upon nanoparticle synthesis. The OCH displayed a mild positive zeta potential of + 11.4 mV, reflecting limited electrostatic repulsion and a potential for aggregation. Conversely, the OCH-AgNPs exhibited a strongly negative zeta potential of − 47.0 mV, suggesting enhanced colloidal stability due to increased surface charge. TEM further confirmed the nanoscale structure and uniformity of the synthesized AgNPs. TEM images revealed well-dispersed, spherical nanoparticles ranging from 10 to 33 nm in diameter. Quantitative size analysis of 55 individual particles demonstrated a unimodal distribution, with a modal diameter of 14–15 nm, comprising 12% of the total particle population. The histogram displayed a near-normal distribution with slight positive skewness, indicating controlled nucleation and growth processes. The mean particle diameter was calculated to be 19.6 ± 6.1 nm, with a coefficient of variation (CV) below 31%, supporting high synthetic reproducibility and moderate mono dispersity. The superimposed Gaussian-smoothed curve clearly delineated the predominant population range (14–17 nm), aligning with optimal sizes for biomedical applications such as drug delivery and diagnostic imaging.

### In vitro biological activities of OCH-AgNPs

#### Antimicrobial evaluation against pathogenic fungal and bacterial strains

**Fig. 5:**
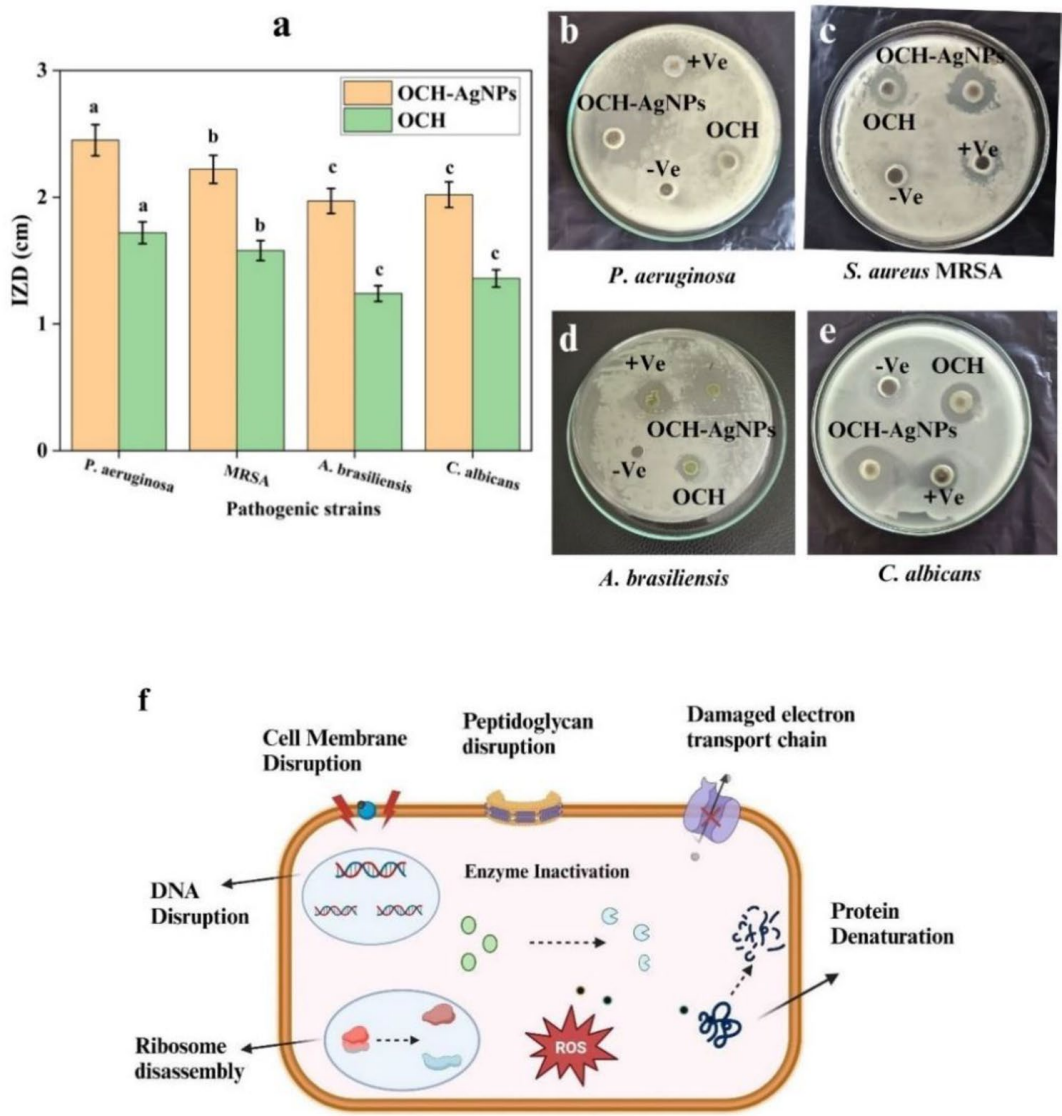
(**a**) Quantitative comparison of inhibition zone diameters (IZD) for OCH and OCH-AgNPs against various microbial strains. Bars not sharing the same lowercase letter indicate statistically significant differences at *p* < 0.05 (one-way ANOVA, Tukey’s post hoc test). (**b**–**e**) Representative agar well diffusion images showing inhibition zones produced by OCH and OCH-AgNPs. (**f**) Schematic illustration of proposed antimicrobial mechanisms of AgNPs, including membrane disruption, oxidative stress generation, DNA interference, and enzymatic inhibition

The antimicrobial activity of silver nanoparticles synthesized from OCH-AgNPs was assessed against a panel of bacterial and fungal pathogens using the agar well diffusion method (Fig. 5). Compared to the crude OCH extract, OCH-AgNPs exhibited significantly greater inhibitory effects across all tested strains. For bacterial pathogens, the inhibition zones increased from 1.72 cm to 2.45 cm against *P. aeruginosa* and from 1.58 cm to 2.21 cm against methicillin-resistant *S. aureus* (MRSA). Similar enhancements were observed in antifungal activity, with inhibition zones increasing from 1.24 cm to 1.97 cm for *A. brasiliensis* and from 1.36 cm to 2.02 cm for *C. albicans*. These increases were statistically significant (*p* < 0.05), confirming the superior antimicrobial efficacy of the nanoparticle formulation. The enhanced activity is attributed to the multifaceted mechanisms of silver nanoparticles, including disruption of microbial membranes, induction of reactive oxygen species (ROS), interference with DNA replication, and inhibition of microbial enzymes, as illustrated in Fig. 5.

### Minimum inhibitory concentration (MIC) analysis

**Fig. 6:**
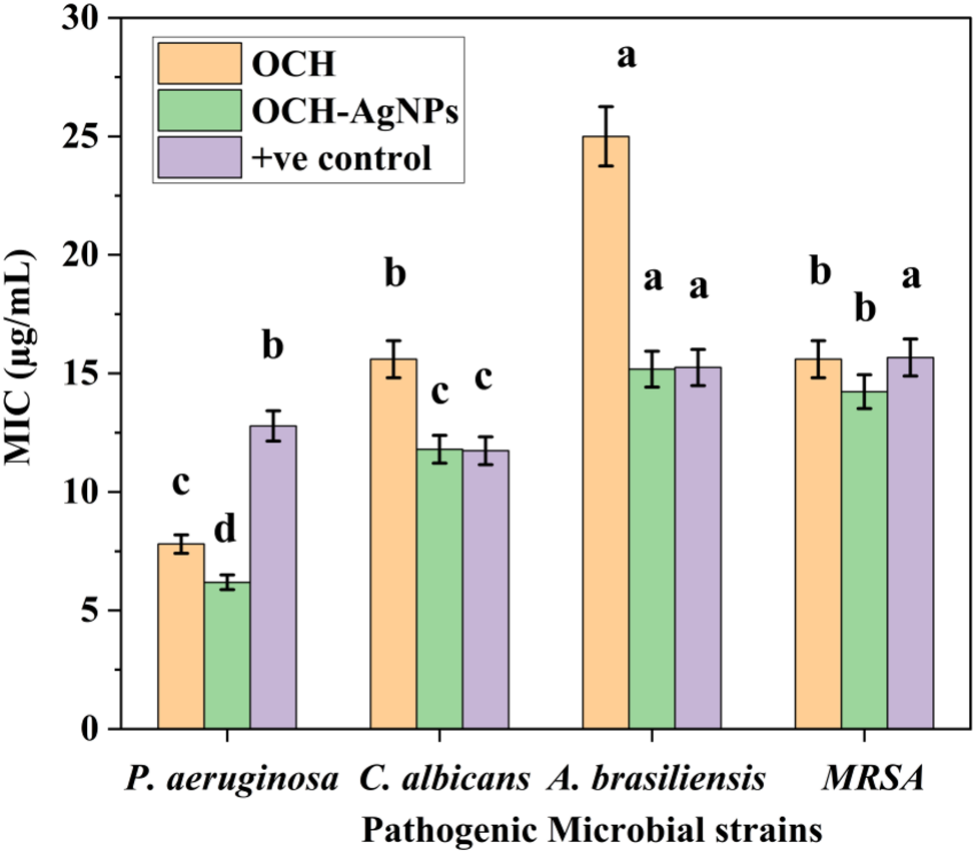
Minimum inhibitory concentrations (MICs) of OCH-AgNPs, OCH, and positive controls (gentamycin at 10 µg for bacteria and fluconazole at 25 µg for yeast and fungi) against pathogenic microbial strains. The same lowercase letter indicate statistically significant differences at *p* < 0.05 (one-way ANOVA, Tukey’s post hoc test).Values represent the mean ± SD from three independent replicates

The MIC values, presented in Fig. 6, demonstrate the enhanced antimicrobial potency of OCH-AgNPs compared to both the crude OCHand standard positive controls. Among the tested pathogens, *P. aeruginosa* showed the highest sensitivity to OCH-AgNPs, with an MIC of 6.19 ± 0.31 µg/mL, significantly lower than that of OCH (7.80 ± 0.39 µg/mL) and the positive control, gentamicin (12.78 ± 0.64 µg/mL) (*p* < 0.05). Against *C. albicans*, OCH-AgNPs exhibited an MIC of 11.08 ± 0.55 µg/mL, which was also significantly lower than the MIC for OCH (15.60 ± 0.78 µg/mL) and fluconazole (25 µg/mL). A similar trend was observed for methicillin-resistant *S. aureus* (MRSA), where OCH-AgNPs achieved an MIC of 14.23 ± 0.71 µg/mL, compared to 15.60 ± 0.78 µg/mL for OCH and 12.78 ± 0.64 µg/mL for gentamicin. Against *A. brasiliensis*, OCH-AgNPs demonstrated substantial antifungal activity with an MIC of 15.18 ± 0.76 µg/mL, markedly lower than the MIC of OCH (25.00 ± 1.25 µg/mL) and comparable to fluconazole (15.25 ± 0.76 µg/mL). These results confirm the superior antimicrobial efficacy of OCH-AgNPs across both bacterial and fungal strains, supporting their potential as effective broad-spectrum antimicrobial agents. All MIC values were determined in triplicate, and statistical significance was evaluated using one-way ANOVA followed by Tukey’s post hoc test (*p* < 0.05).

### Larvicidal activity optimization against *C. pipiens* larvae

#### Box-behnken design optimization

**Fig. 7:**
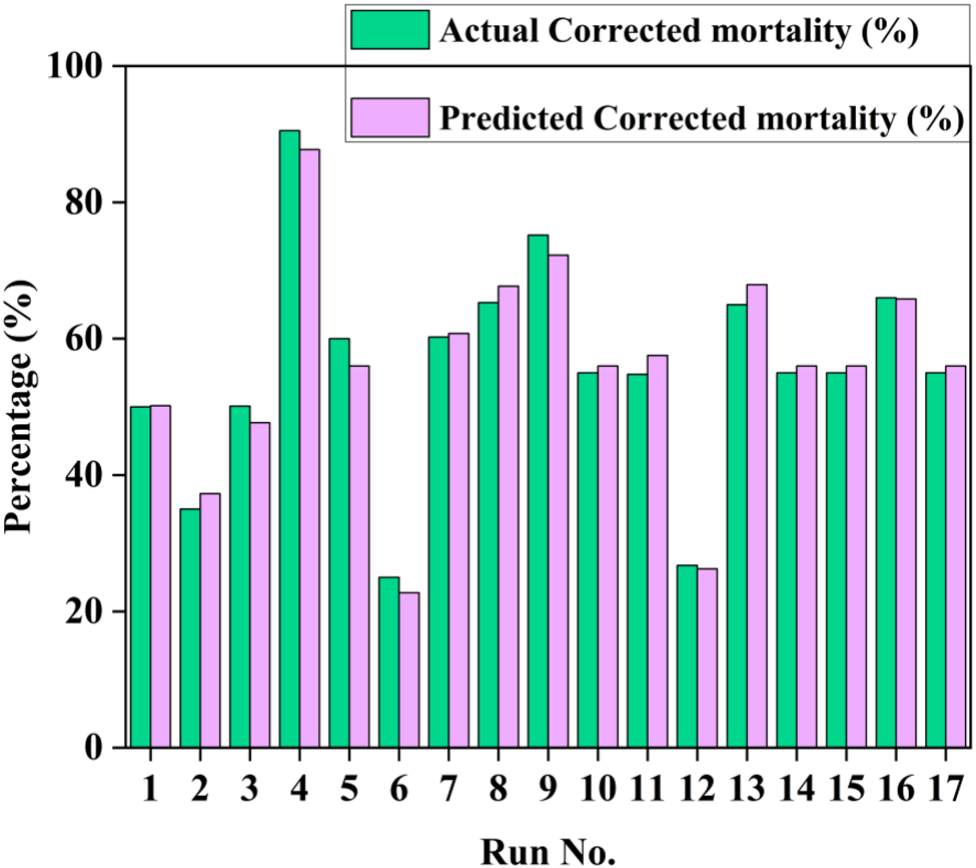
Predicted and actual mortality (%) based on Box-Behnken design optimization for larvicidal activity of OCH against second and third instar *C. pipiens* larvae

**Fig. 8:**
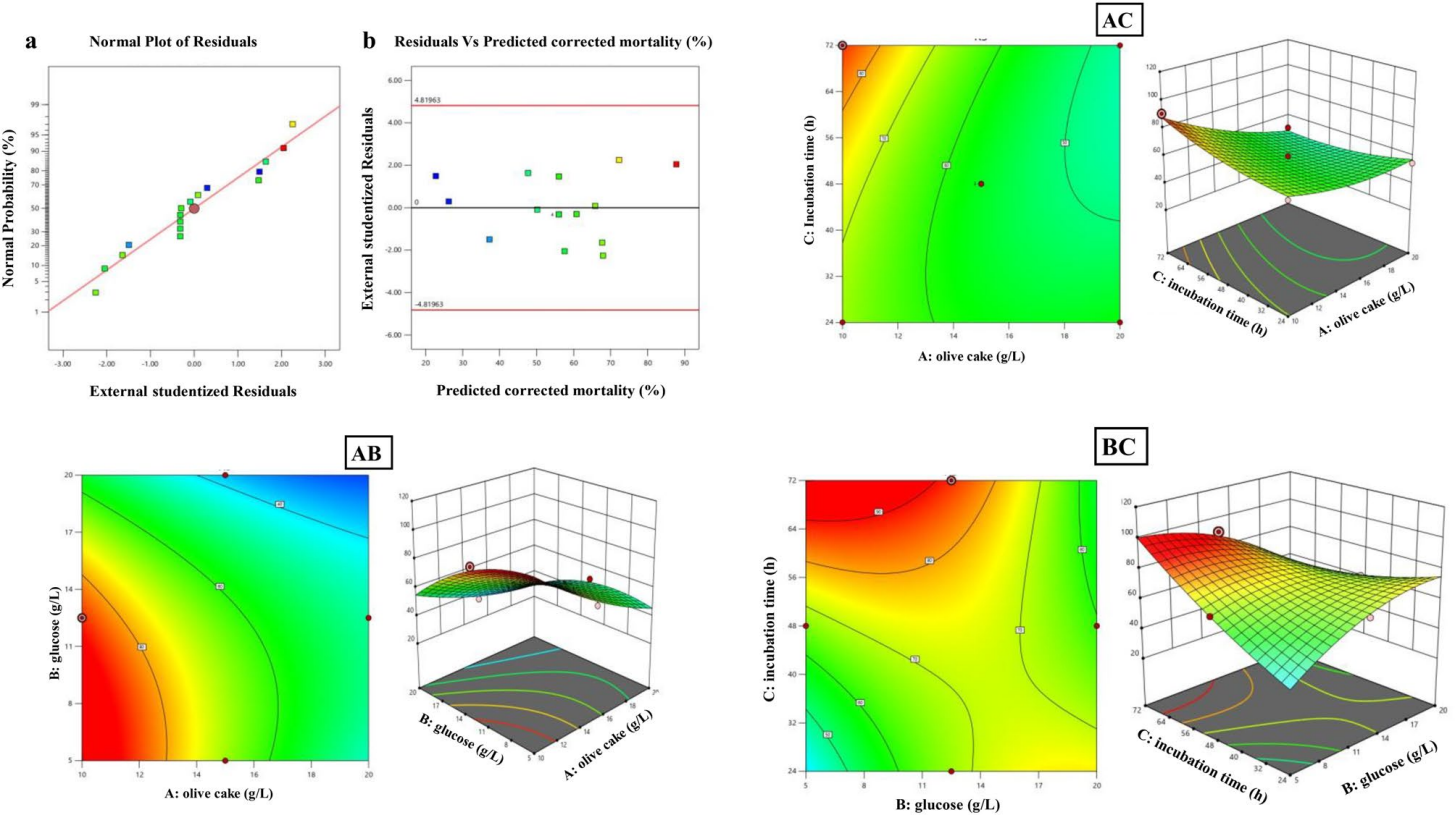
Response surface methodology analysis and model validation for larvicidal corrected mortality (%) optimization using OCH-AgNPs from *P. fluorescence*. (**a**-**b**) Model validation: (**a**) normal probability plot of residuals confirming normality, (**b**) residuals vs. predicted values showing acceptable scatter within limits, validating model adequacy (F = 42.67, *p* < 0.0001). (**c**–**e**) 3D surfaces and contour plots for AB, AC and BC interactions, with AB showing characteristic saddle-shaped response where maximum mortality occurs at medium level of olive cake and glucose

Larvicidal activity of OCH against *C. pipiens* larvae was optimized using a Box-Behnken design incorporating three variables: olive cake concentration (A), glucose concentration (B), and incubation time (C) (Fig. 7). The design revealed significant variability in mortality outcomes based on treatment combinations, with the highest larvicidal efficacy (90.54% actual, 87.76% predicted) observed under conditions of low olive cake, medium glucose, and extended incubation (run 4). Conversely, the lowest efficacy (26.76% actual, 26.24% predicted) occurred with high olive cake and low glucose at moderate incubation (run 12). ANOVA confirmed the model’s significance (F = 42.67, *p* < 0.0001), with all three main factors significantly influencing mortality (F-values: A = 117.62, B = 16.56, C = 4.83; *p* < 0.05), along with notable interaction effects between AB, AC, and BC (F-values: 19.59, 20.87, and 149.96, respectively; *p* < 0.0001). The lack-of-fit test was non-significant (*p* > 0.05), supporting model adequacy. The coded regression equation for predicted mortality (%) was: Y = 56–12.5563 A + 4.71125B + 2.545 C + 7.2475AB − 7.48AC − 20.05BC + 4.94125 A² − 10.1888B² + 4.23875 C², where A, B, and C represent olive cake concentration (g/L), glucose concentration (g/L), and incubation time (h), respectively. Model validation (Fig. 8a–b) showed a near-linear residual normal probability plot and acceptable scatter in residuals vs. predicted plots, confirming normality and homoscedasticity. Response surface and contour plots (Fig. 8c–e) revealed a saddle-shaped interaction between olive cake and glucose levels, with maximum larvicidal efficacy occurring either at low olive cake with high glucose or vice versa, consistent with the significant AB interaction.

### Larvicidal activity of OCH-AgNPs against *C. pipiens* larvae

The larvicidal efficacy of OCH and its silver nanoparticle formulation (OCH-AgNPs) was evaluated against second and third instar *C. pipiens* larvae (Table 3). Results revealed a dose-dependent increase in mortality for both treatments, with OCH-AgNPs demonstrating significantly enhanced toxicity compared to the crude OCH extract. The median lethal concentration (LC₅₀) for OCH-AgNPs was 0.40 µg/mL (95% CI: 0.25–0.55 µg/mL), which was approximately 143-fold lower than that of OCH (57.22 µg/mL; 95% CI: 36.11–85.42 µg/mL). Similarly, the LC₉₀ values were 1.01 µg/mL for OCH-AgNPs and 247.50 µg/mL for OCH. At a concentration of 1.0 µg/mL, OCH-AgNPs achieved 98.86% mortality, while the highest tested concentration of OCH (100 µg/mL) resulted in only 70.25% mortality (*p* < 0.05). Chi-square values for the probit analysis confirmed a good model fit for both treatments (OCH-AgNPs: χ² = 0.320; OCH: χ² = 0.856; *p* > 0.05). These findings demonstrate that biogenic silver nanoparticles synthesized from olive cake hydrolysate possess potent larvicidal activity at significantly lower concentrations, highlighting their potential utility in eco-friendly mosquito control strategies.

**Table 3:** Larvicidal efficacy of OCH and OCH-AgNPs against *C. pipiens* larvae after 24 h exposure

Concentration (µg/mL)	24 h Mortality (%) ± SD	LC₅₀ (µg/mL) (LCL-UCL)	LC₉₀ (µg/mL) (LCL-UCL)*	χ²
OCH		57.22 (36.11–85.42)	247.50 (180.25–380.62)	0.856
Control	00 ± 00^f^	-	-	-
20	18.78 ± 3.35^d^	-	-	-
40	35.39 ± 3.01^c^	-	-	-
60	55.18 ± 5.51^b^	-	-	-
80	58.15 ± 6.08^b^	-	-	-
100	70.25 ± 5.51^a^	-	-	-
OCH -Ag NPs		0.40 (0.25–0.55)	1.01 (0.80–1.45)	0.320
Control	00 ± 00f	-	-	-
0.2	25.68 ± 4.41^e^	-	-	-
0.4	40.00 ± 2.89^d^	-	-	-
0.6	68.74 ± 6.01^c^	-	-	-
0.8	85.15 ± 4.41^b^	-	-	-
1.0	98.86 ± 1.67^a^	-	-	-

LCL: lower confidence limit; UCL: upper confidence limit. Values are expressed as mean ± SD (*n* = 3). Different superscript letters in the same treatment group indicate statistically significant differences (*p* < 0.05)

### Morphological and histopathological effects of OCH-AgNPs on *C. pipiens* larvae

**Fig. 9:**
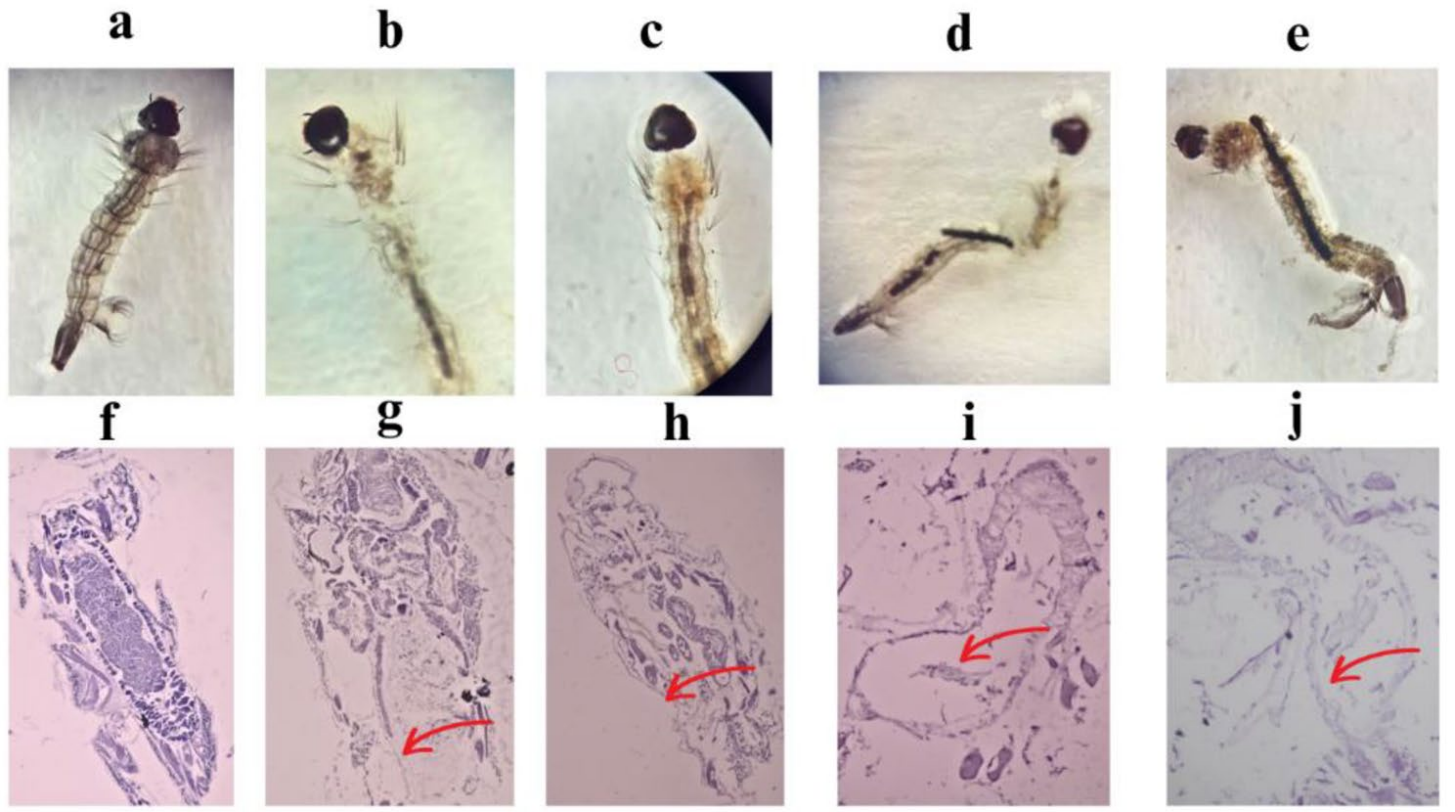
Morphological and histopathological alterations in *C. pipiens* larvae following exposure to OCH-AgNPs. Top row (**a**–**e**): External morphology, (**a**) control larva with normal appearance; (**b**–**e**) increasing malformations with rising OCH-AgNP concentrations, including blackening, transparency loss, and body fragmentation. Bottom row (**f**–**j**): Histological sections of midgut. (**f**) control showing intact epithelial lining; (**g**–**j**) progressive epithelial degeneration, vacuolization, and necrosis. Red arrows indicate areas of severe tissue disruption

Morphological and histopathological assessments (Fig. 9) revealed pronounced external and internal damage in *C. pipiens* larvae treated with OCH-AgNPs. Externally, larvae exposed to increasing concentrations of OCH-AgNPs showed progressive cuticle disruption, body deformation, blackening, and transparency loss, in stark contrast to the intact morphology observed in control larvae. Histological examination of larval midgut sections further confirmed internal tissue damage. Control larvae exhibited well-defined epithelial cell layers and organized midgut architecture. In contrast, larvae exposed to OCH-AgNPs showed dose-dependent epithelial disintegration, cytoplasmic vacuolization, and widespread necrosis. At higher concentrations (0.8–1.0 µg/mL), the midgut structure was almost completely destroyed, indicating irreversible damage likely responsible for larval mortality. These results suggest that OCH-AgNPs exert their larvicidal effect via both external structural disruption and internal histopathological alterations, providing dual mechanisms of toxicity that enhance their efficacy as biological insecticides.

### Biochemical disruption in *C. pipiens* larvae

**Fig. 10:**
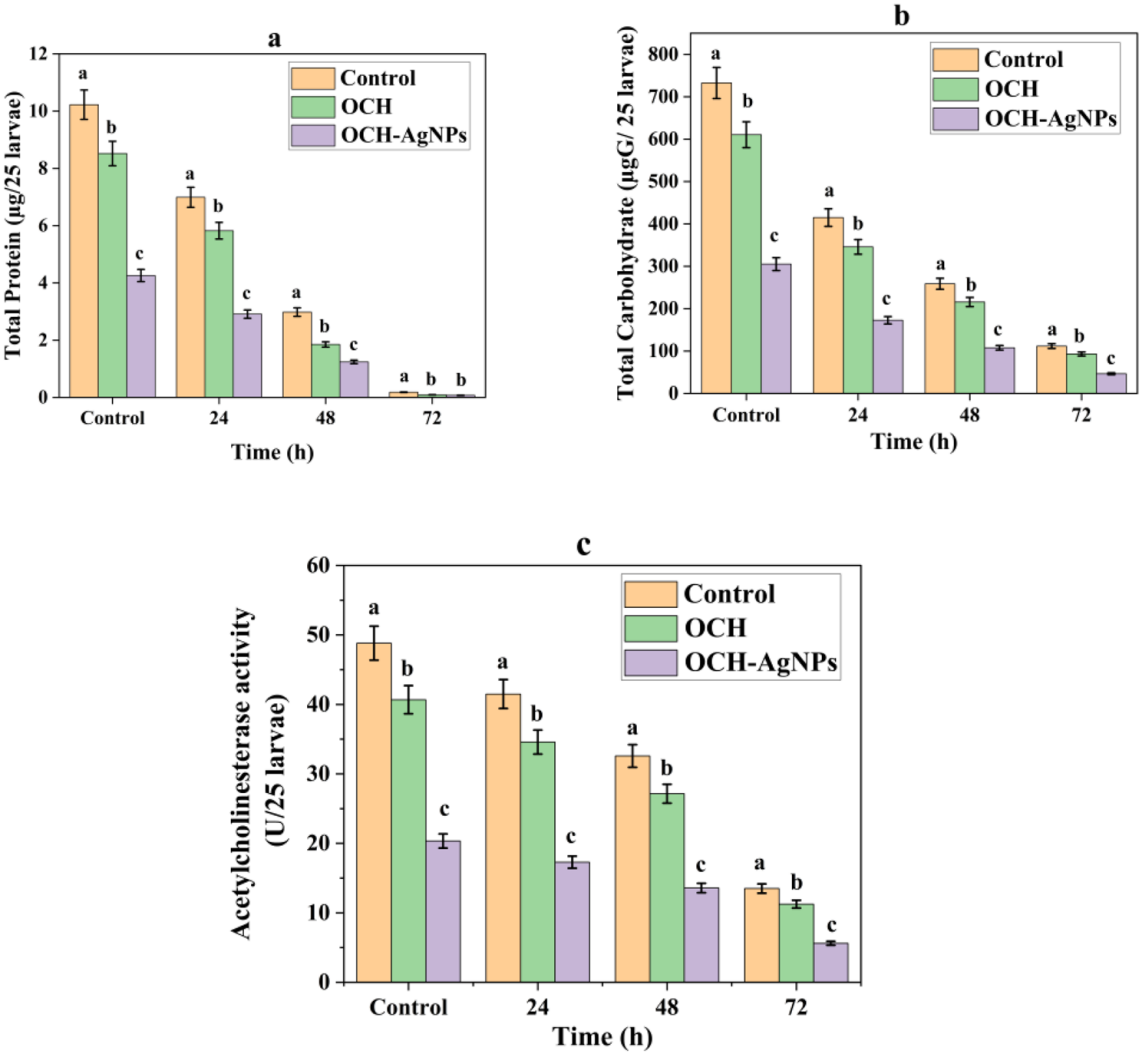
Biochemical analysis: (**a**) Total protein, (**b**) Total carbohydrate, and (**c**) Acetylcholinesterase activity in *C. pipiens* larvae exposed to OCH and OCH-AgNPs. All values are means ± SD (*n* = 3). The same lowercase letter indicate statistically significant differences at *p* < 0.05 (one-way ANOVA, Tukey’s post hoc test).Significant differences (*p* < 0.05) were observed between treatments and the control group at each time point

Biochemical assays (Fig. 10) revealed that both OCH and its silver nanoparticle formulation (OCH-AgNPs) caused significant metabolic and enzymatic disruption in *C. pipiens* larvae, with OCH-AgNPs inducing the most pronounced effects. Total protein levels declined progressively in all treated groups over 72 h. In the control group, protein content decreased from 10.23 µg/25 larvae to 0.18 µg/25 larvae, whereas OCH and OCH-AgNP treatments accelerated this reduction, reaching 0.09 µg/25 larvae and 0.08 µg/25 larvae, respectively. Similarly, carbohydrate content in control larvae decreased from 732.6 µg/25 larvae to 111.78 µg/25 larvae, while OCH-AgNPs caused a steeper depletion, from 305.25 µg/25 larvae to 46.58 µg/25 larvae, indicating substantial metabolic impairment.

Acetylcholinesterase (AChE) activity, essential for neural function, was markedly inhibited following OCH-AgNP exposure. In the control, AChE activity dropped from 48.82 U/25 larvae to 13.50 U/25 larvae, while OCH-AgNPs reduced it from 20.34 to 5.63 U/25 larvae within the same period. All differences were statistically significant (*p* < 0.05). These findings confirm that OCH-AgNPs disrupt both energy metabolism and neural function in mosquito larvae, likely contributing to their enhanced larvicidal potency through combined metabolic suppression and neurotoxicity.

### Molecular docking insights: mode of action

**Fig. 11:**
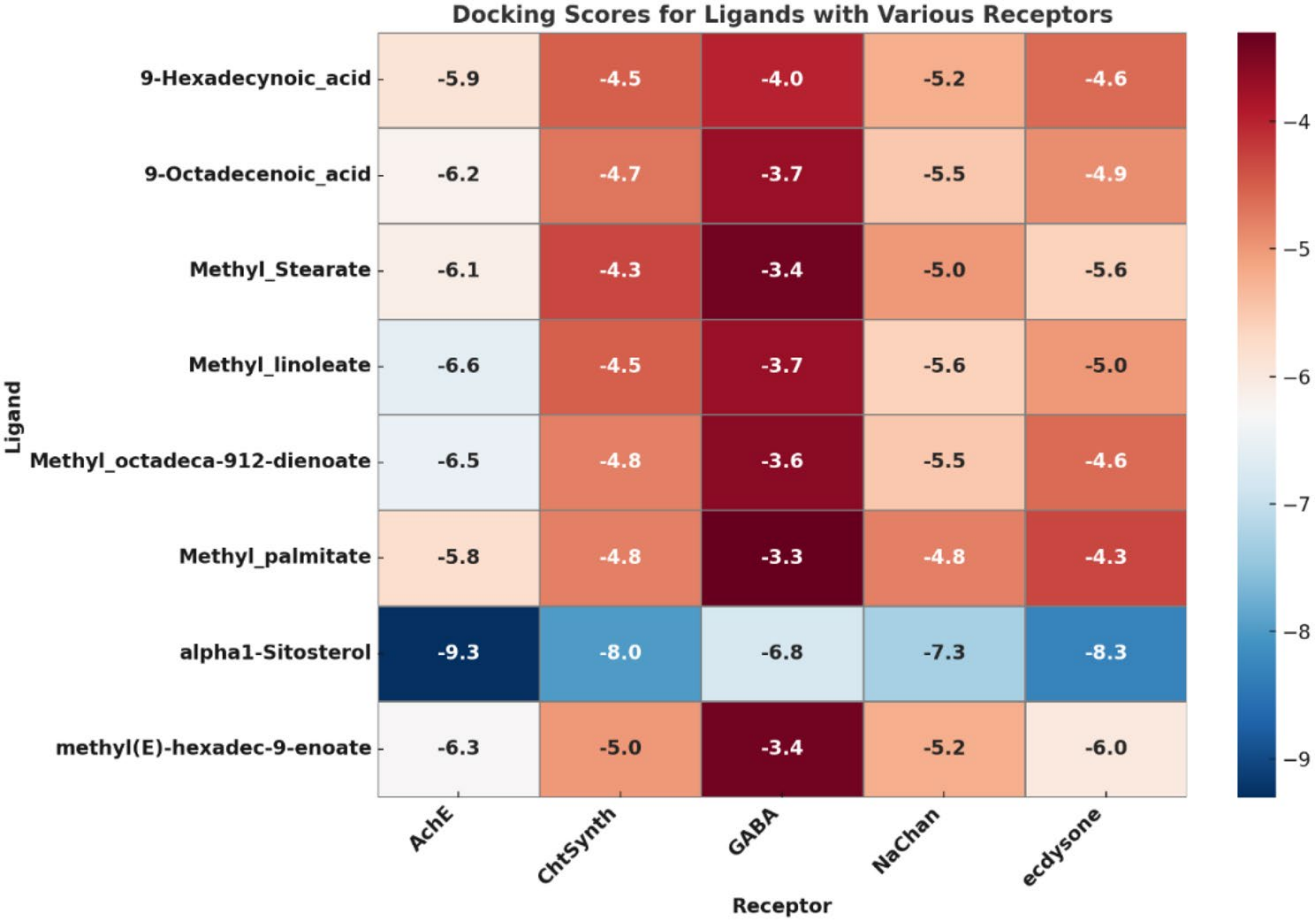
Heat map illustrating molecular docking binding affinities (kcal/mol) of selected ligands from OCH and OCH-AgNPs against larval metabolic and neurological targets: acetylcholinesterase (AChE), sodium channels, chitin synthase, and GABA receptors. Stronger binding (more negative values) indicates higher affinity

**Fig. 12:**
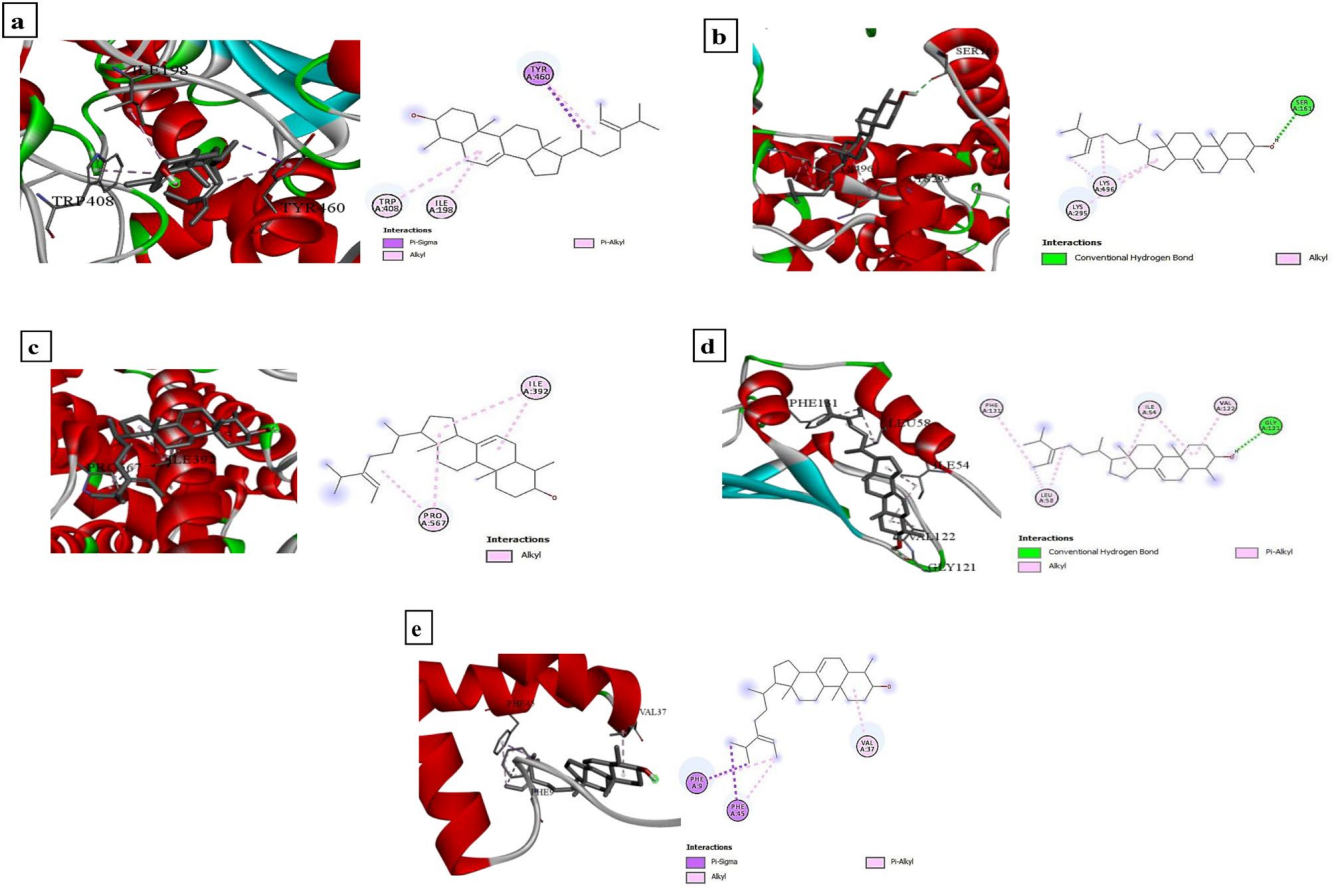
Docking view of Alpha-sitosterol produced by *P. fluorescens* (**a**) AchE_Q86GC8 (**b**) AchE_Q86GC8 (**c**) ChtSynth_A0A2R4SD28 (**d**) GABA_A0A1 × 9PRB2 (**e**) NaChan_D2XKJ6. The three-dimensional interaction diagrams are positioned on the left side, while the two-dimensional complex structures are displayed on the right. All evaluated compounds appear in grey ball-and-stick representation, with various interaction bonds illustrated by differently colored dashed lines. These visualizations were generated using BIOVIA CLINT 2020 software

Molecular docking studies (Figs. 11 and 12) supported the biochemical results by revealing strong interactions between bioactive compounds derived from OCH and key larval molecular targets. Among the eight tested ligands, α-sitosterol exhibited the highest binding affinity, particularly toward acetylcholinesterase (AChE), with a binding energy of − 9.3 kcal/mol, suggesting a strong inhibitory potential. AChE consistently showed the strongest ligand binding across the target panel (binding scores ranging from − 5.8 to − 9.3 kcal/mol), followed by moderate affinity for sodium channels, chitin synthase, and GABA receptors. In contrast, GABA receptors displayed comparatively lower affinities (− 3.3 to − 6.8 kcal/mol), indicating secondary roles in larval toxicity. Fatty acid methyl esters such as methyl linoleate and methyl octadeca-9,12-dienoate demonstrated moderate binding affinities, whereas methyl palmitate showed weaker interactions. Structural visualization (Fig. 12) revealed that α-sitosterol formed diverse non-covalent interactions with all key targets. In AChE, it engaged in Pi–Sigma and Pi–Alkyl interactions with TYR460, ILE198, and TRP408. In sodium channels, Pi–Sigma and Alkyl bonds were observed with PHE9, PHE45, and VAL37, while a hydrogen bond (2.31 Å) formed with GLY121 in the GABA receptor, along with multiple hydrophobic interactions. These results suggest that α-sitosterol exerts multifunctional effects, targeting both metabolic enzymes and neuronal receptors, thereby reinforcing its potential role in the observed larvicidal activity of OCH-AgNPs.

### Cytotoxicity on human skin fibroblasts (HSF)

**Fig. 13:**
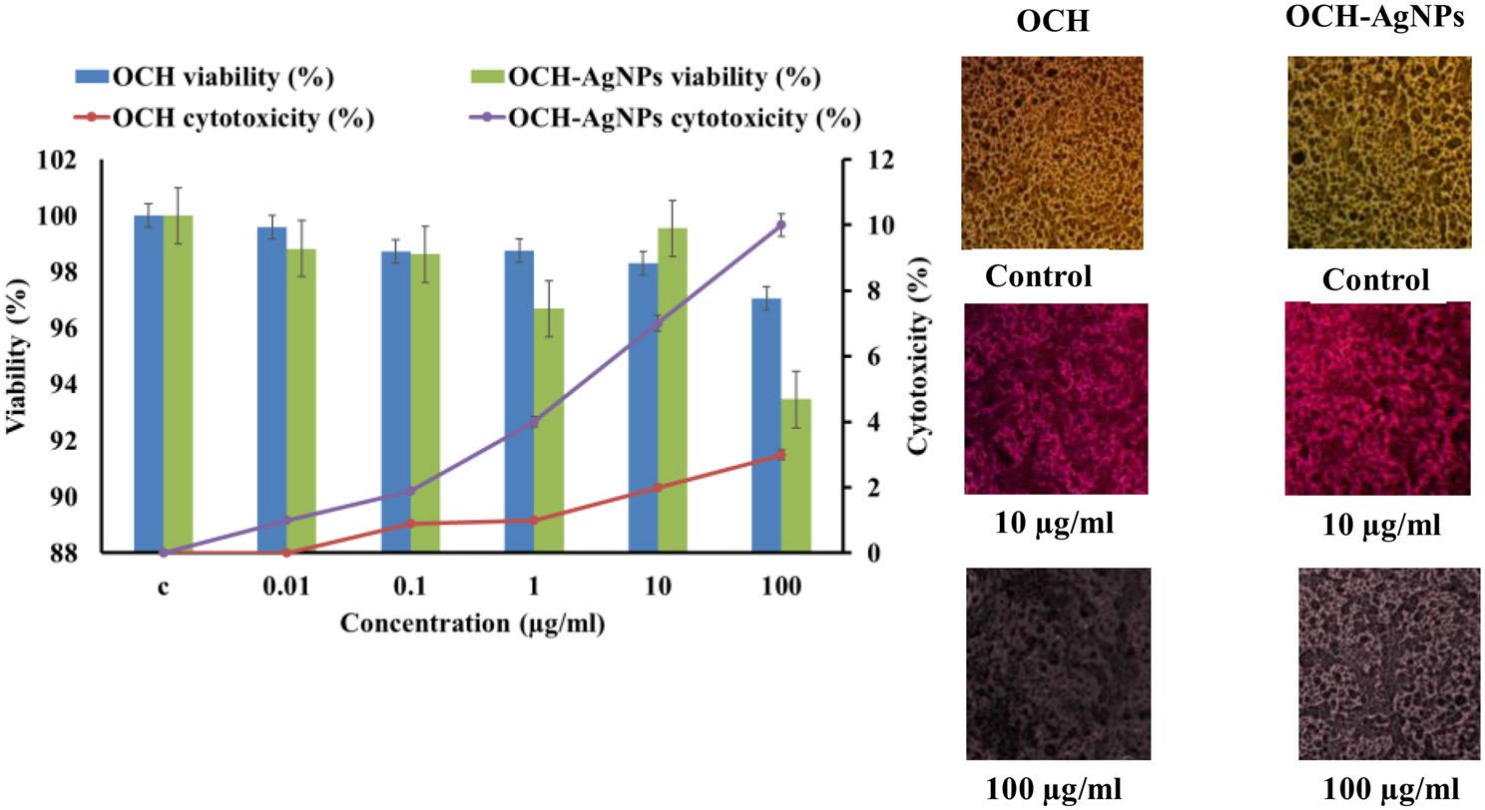
Cytotoxicity and cell viability assessment of olive cake hydrolysate (OCH) and silver nanoparticle-enhanced OCH (OCH-AgNPs) on normal cell lines. Left panel shows concentration-dependent effects on cell viability and cytotoxicity for both treatments. Right panel displays microscopic images of cell morphology at different concentrations: control cells show normal dense growth patterns, 10 µg/ml treatments maintain cellular integrity with slight color changes, while 100 µg/ml exposures result in visible cell damage and reduced density, with OCH-AgNPs showing more pronounced cytotoxic effects than OCH alone. The data represent mean values of three replicates ± SD

Cytotoxicity assays (Fig. 13) were conducted to evaluate the biocompatibility of OCH and OCH-AgNPs on normal human skin fibroblast (HSF) cells. Both treatments demonstrated concentration-dependent effects on cell viability. At low concentrations (0.01–0.1 µg/mL), both OCH and OCH-AgNPs maintained cell viability above 98%, with cytotoxicity below 2%, indicating excellent biocompatibility at biologically relevant larvicidal doses. However, at higher concentrations (10–100 µg/mL), a dose-dependent reduction in viability was observed, particularly for OCH-AgNPs. At 100 µg/mL, OCH-AgNPs reduced viability to approximately 90%, while OCH alone retained over 95% viability. Microscopic observation using MTT staining confirmed these findings. Control and low-dose treated cells showed dense monolayers with uniform morphology and intense pink staining. At 100 µg/mL, cells exposed to OCH-AgNPs exhibited visible morphological changes, including reduced confluence and loss of staining intensity, indicating membrane damage and cytotoxic stress. These findings suggest that while OCH-AgNPs are safe at effective larvicidal concentrations, caution should be exercised at elevated doses to avoid off-target cytotoxic effects.

## Discussion

The GC-MS analysis revealed substantial chemical transformation of olive cake following P. *fluorescens* degradation, with the emergence of bioactive compounds such as 24-Norursa-3,12-diene (21.96%) and 9,12-Octadecadienoyl chloride ((9Z,12Z)-Octadeca-9,12-dienoyl chloride) (18.83%). These findings align with previous studies by Abu-Hussien et al. (2021) [17], who reported similar enzymatic conversions in microbial degradation of agricultural waste, though our results demonstrate higher concentrations of terpenoid derivatives.

The detection of 24-Norursa-3,12-diene is particularly significant, as triterpenoids have been documented to exhibit insecticidal properties [36, 37] against *C. quinquefasciatus* with LC₅₀ values below 5 µg/mL [38, 39]. Compared to conventional chemical extraction methods that typically yield 5–10% terpenoid content, our microbial approach achieved over 20%, suggesting superior bioactive compound liberation through enzymatic pathways.

The metabolic versatility of *P. fluorescens* in converting fatty acid methyl esters into reactive derivatives demonstrates its potential for valorizing agro-waste, consistent with findings by Sharma et al. (2022) [40] who reported similar metabolic profiles in *Pseudomonas*-mediated bioconversion. However, our study extends these findings by quantifying specific bioactive metabolites and directly correlating them with larvicidal efficacy, providing a more comprehensive understanding of the biotransformation process.

The formation of silver nanoparticles (AgNPs) using *P. fluorescens*-derived OCH was visually and spectroscopically confirmed, highlighting the effectiveness of agro-industrial waste as a substrate in green nanoparticle synthesis. The observed color change from yellowish-green to dark brown upon the addition of silver nitrate is a well-documented indicator of AgNP formation, attributed to the excitation of surface plasmon resonance (SPR) caused by collective oscillation of conduction electrons on the nanoparticle surface [41]. This transformation not only signifies the reduction of Ag⁺ ions to elemental silver (Ag⁰) but also reflects the presence of reducing agents in the hydrolysate, likely microbial metabolites including phenolics, flavonoids, and organic acids generated during fermentation.

UV–Visible spectroscopy further validated the synthesis, with a distinct SPR absorption peak at approximately 420 nm in the OCH-AgNPs sample, consistent with the typical optical signature of spherical silver nanoparticles [24]. This peak was absent in the untreated OCH, which exhibited a broader, less defined absorbance around 300 nm likely due to the native aromatic and phenolic compounds present in the olive cake matrix [27].

The FTIR, XRD, and DLS analyses confirmed successful AgNP synthesis with characteristics favorable for biological applications. The observed decrease in hydroxyl band intensity (3300–3500 cm⁻¹) and carbonyl peak shifts align with previous biosynthesis studies by Jatoi et al. (2024) [41] who reported suggest that hydroxyl, carbonyl, and possibly amine functional groups played a central role in both reducing Ag⁺ ions and stabilizing the synthesized AgNPs and the shift confirms effective nanoparticle formation and stabilization, likely facilitated by the reducing and capping properties of OCH-derived biomolecules.

Though our OCH-mediated synthesis demonstrated more pronounced spectral changes, suggesting stronger biomolecule-nanoparticle interactions. The XRD pattern, showing characteristic FCC silver peaks at 2θ = 38°, 44°, 64°, and 77°, matches reports by Asif et al. (2022) [42], but our crystallite size calculation (10–50 nm range) falls within the optimal range for larvicidal applications, superior to many plant-mediated syntheses that often produce larger particles (> 100 nm).

The DLS results showing size reduction from 894.8 nm (OCH) to 109.8 nm (OCH-AgNPs) with improved colloidal stability (zeta potential: -47 mV) compare favorably with studies by Soliman et al. (2024) [43], who achieved similar size distributions using conventional plant extracts. They reports the high absolute value is indicative of effective electrostatic repulsion, most likely conferred by negatively charged functional groups from the OCH matrix bound to the nanoparticle surface. The resulting charge stabilization reduces aggregation risk and contributes to long-term dispersion in aqueous environments. However, our approach demonstrates superior stability parameters, likely due to the diverse biomolecule profile generated through microbial processing. The observed discrepancy between DLS and TEM sizes is attributed to the hydrated shell and possible mild aggregation effects in DLS, which reflect the hydrodynamic diameter, compared to the dry-state measurement by TEM Jatoi et al. (2024) [41].

The antimicrobial efficacy of OCH-AgNPs showed remarkable enhancement compared to OCH alone, with P. aeruginosa demonstrating the greatest sensitivity. These results corroborate findings by Bhat et al. (2021) [44], who reported preferential activity against Gram-negative bacteria due to thinner peptidoglycan layers. However, our MIC values were consistently lower than those reported for conventional AgNPs, suggesting that the microbially-derived capping agents provide additional antimicrobial benefits [45]. The activity against MRSA positions OCH-AgNPs as a promising alternative to conventional antibiotics, addressing the critical need for new antimicrobial agents highlighted by the WHO priority pathogen list.

Compared to standard antimicrobial agents, our OCH-AgNPs demonstrated broader spectrum activity with reduced resistance potential, consistent with the multi-target mechanism proposed by Nayaka et al. (2020) [46]. This advantage over single-target conventional antibiotics represents a significant advancement in antimicrobial technology.

The Box-Behnken optimization revealed that low olive cake concentration, moderate glucose levels, and prolonged incubation maximized larvicidal activity (90.54% mortality), contrasting with intuitive expectations. This inverse relationship between substrate concentration and bioactivity suggests substrate inhibition effects, as previously observed by Mohamed Korayem et al. (2025) [16] in similar bioprocessing systems. However, our findings extend beyond previous work by quantifying optimal parameter combinations and achieving higher mortality rates than reported in comparable studies.

The exceptional enhancement achieved through nanoparticle synthesis (LC₅₀: 0.40 µg/ml vs. 57.22 µg/ml for OCH alone) represents a 143-fold improvement, surpassing the 10–50 fold enhancements typically reported in green larvicide studies [47–49]. This positions OCH-AgNPs among the most potent eco-friendly larvicidal agents documented, with efficacy comparable to synthetic pesticides but with superior environmental compatibility.

The comprehensive biochemical disruption observed in treated larvae including 88.5% reduction in acetylcholinesterase activity, complete protein depletion, and carbohydrate exhaustion provides mechanistic insight into the rapid mortality achieved. These findings align with molecular docking results showing strong α-sitosterol binding to AChE (-9.3 score), confirming neurotoxic mechanisms. Compared to conventional organophosphate insecticides that primarily target AChE, our OCH-AgNPs demonstrate multi-target activity affecting neurological, metabolic, and developmental pathways simultaneously.

The molecular docking predictions, while not experimentally validated, suggest interactions with multiple receptor systems including chitinase synthase and ecdysone receptors, indicating developmental disruption mechanisms. This multi-target approach reduces resistance development risk compared to single-target conventional insecticides, as demonstrated by Mansour et al. (2023) [20].

The cytotoxicity analysis on human skin fibroblasts demonstrated favorable safety compared to conventional larvicides such as temephos and diflubenzuron, which show significant cytotoxic effects at comparable concentrations [20]. The biodegradable nature of OCH-derived components provides additional environmental advantages over persistent synthetic pesticides, addressing growing concerns about ecological contamination.

### Study limitations and future directions

While this study demonstrates significant potential for OCH-AgNPs in vector control applications, several limitations must be addressed for practical implementation. The controlled laboratory conditions may not reflect real-world environmental variability affecting nanoparticle stability and efficacy, necessitating comprehensive field trials under diverse climatic and water quality conditions. The molecular docking predictions require experimental validation through in vitro receptor binding assays, while the cytotoxicity assessment limited to human skin fibroblasts needs expansion to multiple cell lines and animal models for regulatory compliance. Future research should prioritize field validation studies incorporating variable environmental parameters, comprehensive toxicological evaluation across diverse biological systems, resistance monitoring through multi-generation mosquito exposure studies, and environmental fate assessment to determine nanoparticle persistence and bioaccumulation potential. Advanced developments should focus on formulation optimization for practical application through microencapsulation or controlled-release systems, scale-up feasibility studies examining production economics and cost-effectiveness, integration with existing vector control programs, and expansion to other disease vectors including *Aedes aegypti* and *Anopheles gambiae*. Additionally, investigating smart delivery mechanisms such as pH-responsive targeting systems and combination approaches with biological control agents could enhance selectivity and efficacy while minimizing environmental impact, ultimately facilitating the translation of this promising green nanotechnology from laboratory proof-of-concept to sustainable field application.

## Conclusion

This study demonstrates the successful biosynthesis of silver nanoparticles (OCH-AgNPs) through *P. fluorescens*-mediated transformation of olive cake hydrolysate, offering an eco-friendly and sustainable approach to mosquito control. The biosynthesized AgNPs showed potent larvicidal and antimicrobial activity, while maintaining low cytotoxicity to normal human cells. These findings highlight the dual biological potential and biosafety of OCH-AgNPs. By integrating microbial bioprocessing with agricultural waste valorization, this work contributes to the development of sustainable nanobiotechnology-based tools for public health and environmental applications. Future efforts should focus on large-scale production, formulation optimization, and field-level implementation. This eco-compatible strategy presents a viable pathway for turning agro-industrial waste into high-value biopesticides with potential applications in sustainable public health management.

## Electronic supplementary material

Below is the link to the electronic supplementary material.

Supplementary Material 1

## Data Availability

The raw data and analysed data used during the current study are available from the corresponding author upon reasonable request. All microbial pathogens were provided by the Nawah scientific (https://nawah-scientific.com/), Cairo, Egypt, and were deposited in the following strain providers: 1- P. aeruginosa ATCC 27853. https://www.atcc.org/products/27853. 2- MRSA. https://www.atcc.org/products/43300. 3- A. brasiliensis ATCC16404. https://www.atcc.org/products/16404. 4- C. albicans ATCC 10231. https://www.atcc.org/products/10231.
