# Supplementary material for: Biogenic silver nanoparticles synthesized from Pseudomonas fluorescens-mediated olive cake waste: antimicrobial, larvicidal activity against Culex pipiens and cytotoxicity assessment
Source: BMC Biotechnol. 2025 Jul 21;25:75. doi: 10.1186/s12896-025-01011-2 (PMC12278677; doi:10.1186/s12896-025-01011-2)
Supplement: Supplementary file 1 — Supplementary Material 1 [file 12896_2025_1011_MOESM1_ESM.pdf]

Biogenic Silver Nanoparticles Synthesized from *Pseudomonas fluorescens*-Mediated Olive Cake Hydrolysate: Antimicrobial, Larvicidal, and Toxicity assessment

Samah H. Abu-Hussien[1], Muhammad Aslam Khan[2], Ammar AL-Farga[3], Ahmed G. Soliman[4], Salwa M. El-Sayed[4], Eslam Adly[5]

[1]Department of Agriculture Microbiology, Faculty of Agriculture, Ain Shams University, 11241,Cairo, Egypt

[2]Department of Biological Sciences, Faculty of Sciences, International Islamic University (IIU), Islamabad, Pakistan

[3]Department of Biochemistry, Faculty of Science University of Jeddah, Saudi Arabia.

[4]Department of Agriculture Biochemistry, Faculty of Agriculture, Ain Shams University, 11241,Cairo, Egypt

[5]Department of Entomology, Faculty of Science, Ain Shams University, Cairo, Egypt

Correspondence: Samah H. Abu-Hussien (samah_hashem1@agr.asu.edu.eg)

a

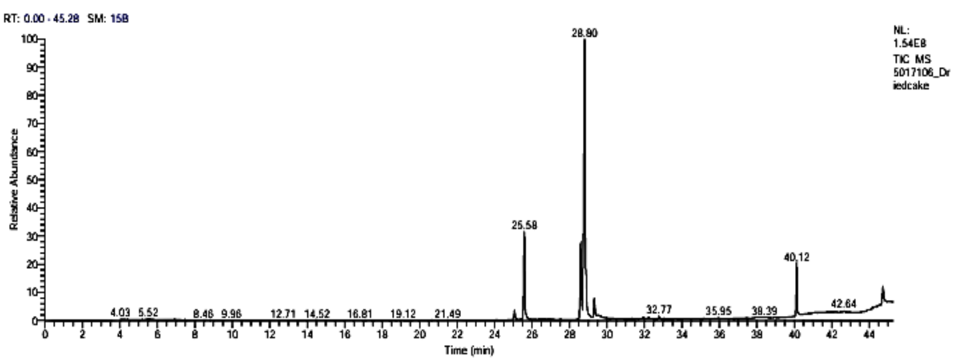

b

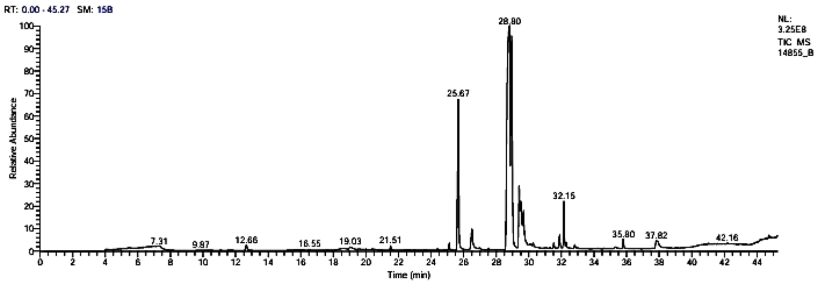

Figure (S1): GC-MS profile of olive cake waste (a) before and (b) after microbial degradation by *P. fluorescens*
